# Permeability of the HIV-1 capsid to metabolites modulates viral DNA synthesis

**DOI:** 10.1371/journal.pbio.3001015

**Published:** 2020-12-17

**Authors:** Chaoyi Xu, Douglas K. Fischer, Sanela Rankovic, Wen Li, Robert A. Dick, Brent Runge, Roman Zadorozhnyi, Jinwoo Ahn, Christopher Aiken, Tatyana Polenova, Alan N. Engelman, Zandrea Ambrose, Itay Rousso, Juan R. Perilla

**Affiliations:** 1 Department of Chemistry and Biochemistry, University of Delaware, Newark, Delaware, United States of America; 2 Pittsburgh Center for HIV Protein Interactions (PCHPI), University of Pittsburgh School of Medicine, Pittsburgh, Pennsylvania, United States of America; 3 Department of Microbiology and Molecular Genetics, University of Pittsburgh School of Medicine, Pittsburgh, Pennsylvania, United States of America; 4 Department of Physiology and Cell Biology, Ben-Gurion University of Negev, Beer Sheva, Israel; 5 Department of Cancer Immunology and Virology, Dana-Farber Cancer Institute, Boston, Massachusetts, United States of America; 6 Department of Medicine, Harvard Medical School, Boston, Massachusetts, United States of America; 7 Department of Molecular Biology and Genetics, Cornell University, Ithaca, New York, United States of America; 8 Department of Structural Biology, University of Pittsburgh School of Medicine, Pittsburgh, Pennsylvania, United States of America; 9 Department of Pathology, Microbiology and Immunology, Vanderbilt University Medical Center, Nashville, Tennessee, United States of America; Loyola University Chicago, UNITED STATES

## Abstract

Reverse transcription, an essential event in the HIV-1 life cycle, requires deoxynucleotide triphosphates (dNTPs) to fuel DNA synthesis, thus requiring penetration of dNTPs into the viral capsid. The central cavity of the capsid protein (CA) hexamer reveals itself as a plausible channel that allows the passage of dNTPs into assembled capsids. Nevertheless, the molecular mechanism of nucleotide import into the capsid remains unknown. Employing all-atom molecular dynamics (MD) simulations, we established that cooperative binding between nucleotides inside a CA hexamer cavity results in energetically favorable conditions for passive translocation of dNTPs into the HIV-1 capsid. Furthermore, binding of the host cell metabolite inositol hexakisphosphate (IP_6_) enhances dNTP import, while binding of synthesized molecules like benzenehexacarboxylic acid (BHC) inhibits it. The enhancing effect on reverse transcription by IP_6_ and the consequences of interactions between CA and nucleotides were corroborated using atomic force microscopy, transmission electron microscopy, and virological assays. Collectively, our results provide an atomistic description of the permeability of the HIV-1 capsid to small molecules and reveal a novel mechanism for the involvement of metabolites in HIV-1 capsid stabilization, nucleotide import, and reverse transcription.

## Abstract 2

This study shows that the HIV-1 capsid protein, in addition to its structural role, regulates reverse transcription, an essential metabolic process of the virus, by mediating the import of nucleotides. In addition, host cell metabolites such as inositol phosphates are recruited by the capsid to regulate viral DNA synthesis.

## Introduction

Fusion between HIV-1 virions and target cells engenders the release of the viral capsid into the host cell cytoplasm. The capsid, a cone-shaped protein assembly composed of approximately 250 capsid protein (CA) hexamers and 12 CA pentamers, encapsulates 2 copies of the viral single-stranded RNA (ssRNA) genome and viral proteins, including reverse transcriptase (RT) and integrase (IN) [1–3]. Successful infection requires reverse transcription of the ssRNA into double-stranded viral DNA (vDNA) and integration of vDNA into the host cell genome. Based on steric hindrance of the intact capsid by the nuclear pore complex [4], major structural rearrangement or capsid shedding has been postulated to occur prior to or during nuclear import [5,6], a process commonly referred to as uncoating. While reverse transcription likely initiates within the capsid and is affected by uncoating, the mechanisms connecting the 2 viral processes remain a long-standing question in HIV-1 biology [7–10].

Inositol phosphates (IPs) are abundant cellular metabolites that play crucial roles in fundamental cellular processes [11]. Interestingly, ions and small molecules like inositol hexakisphosphate (IP_6_) have been reported to bind to the HIV-1 Gag polyprotein and CA hexamers, affecting virion assembly and reverse transcription [4,12–16]. It has been proposed that IP_6_ stabilizes the core by interacting with positively charged residues located near the central axis of a CA hexamer. Nevertheless, CA hexamers have been shown to be stable in the absence of IPs. In addition, we and others have proposed that reverse transcription mechanically induces changes in capsid morphology and triggers uncoating [17,18]. To support vDNA synthesis, RT requires adequate concentrations of deoxynucleotide triphosphates (dNTPs) within the lumen of the capsid. The HIV-1 capsid has been described as semipermeable and proposed to regulate the passage of ions including dNTPs from the cytoplasm to its interior [3,4,8].

To test the hypothesis that IPs and other metabolites modulate the translocation of dNTPs through CA oligomers, we have employed a multipronged approach combining all-atom molecular dynamics (MD) simulations [19], atomic force microscopy (AFM), transmission electron microscopy (TEM), confocal microscopy, and virus infectivity assays. Additionally, we have analyzed the effect of IP_6_ on HIV-1 capsid stability and reverse transcription and determined the molecular mechanism that regulates translocation of dNTPs through the capsid.

## Results and discussion

**Fig 1:**
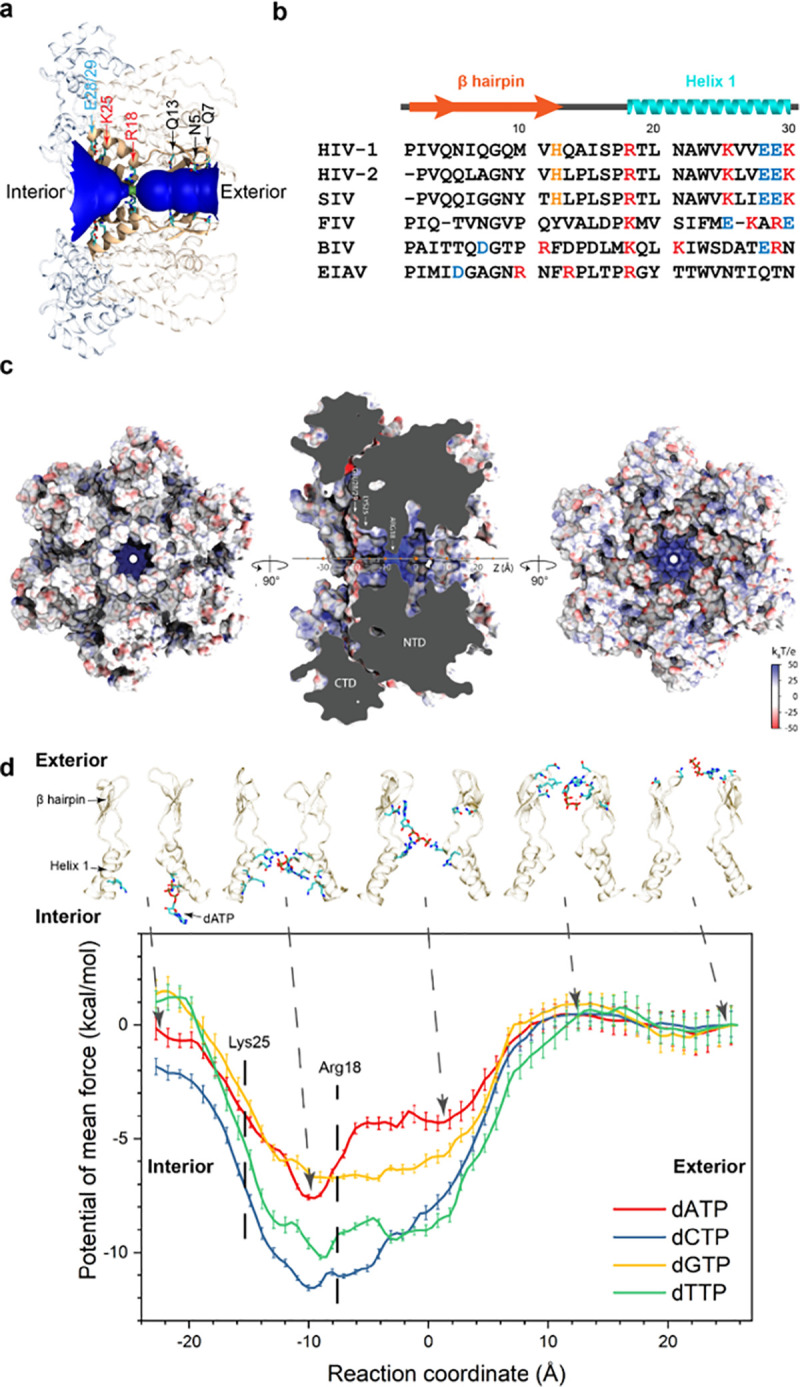
Architecture of the HIV-1 CA hexameric cavity. (a) The radial profile of the native hexamer (PDB accession code 4XFX) showing positions of key CA residues that constitute the inner surface of the central cavity. The interior and exterior of the capsid are located on the left and right of the diagram, respectively. (b) Comparative analysis and sequence conservation of 30 N-terminal CA residues from 6 lentiviruses (HIV-1, HIV-2, SIV, FIV, BIV, and EIAV). Secondary structures are indicated above the sequences. (c) Interior surface, side and exterior surface representing the electrostatic potential. The reaction coordinate (progress variable) for all US/HREX simulations was defined as the position of the center of mass of the nucleotide on the cavity axis. (d) The 1D free energy landscapes of single dNTP translocations through the CA hexamer central cavity. Five representative dATP snapshots are shown to pictorially indicate its interactions with the CA pore. Numerical data for panel D can be found in S1 Data. BIV, bovine immunodeficiency virus; CTD, C terminal domain; dATP, deoxyadenosine triphosphate; dCTP, deoxycytidine phosphate; dGTP, deoxyguanosine triphosphate; dNTP, deoxynucleotide triphosphate; dTTP, deoxythymidine triphosphate; EIAV, equine infectious anemia virus; FIV, feline immunodeficiency virus; NTD, N terminal domain; SIV, simian immunodeficiency virus.

The CA hexameric cavity is surrounded by 6 copies of a β-hairpin, helix 1, and a short loop connecting them (Fig 1A and 1B). Its radial profile, illustrated in Fig 1A for the native CA hexamer [20], contains an exterior tubular channel and an interior conical volume separated by a 1.4 Å radial constriction near the Arg18 residues (Fig 1A). The inner surface of the outward facing cavity is surrounded by residues Asn5, Gln7, and Gln13 that lie within or nearby the β-hairpin. Residues within helix 1 are nearly identical across primate lentiviruses, including invariant charged residues Arg18, Lys25, Glu28, Glu29, and Lys30 (Fig 1B). These residues, which account for the electrostatic potential of the hexamer cavity, contrast to the negative charge found in the exposed surfaces of the hexamer (Fig 1C). Overall, based exclusively on the structural features of CA hexamers, the volume occupied by the ring of 6 Arg18 side chains represents a steric barrier for translocation of small molecules through the central cavity.

To explore the effects of the dynamic behavior of CA hexamers on the permeability of molecules through assembled HIV-1 capsids, we employed free-energy MD simulations to determine the free energy landscapes of dNTPs and IP_6_ interacting with the central cavity (Fig 1D, S1 Fig, S1 Table). Since carboxybenzenes like benzenehexacarboxylic acid (BHC, or mellitic acid) were previously reported to reduce endogenous reverse transcription [8], their free energy landscapes were also calculated in the present study. To describe the location of the nucleotide in the cavity, a progress variable (PV) was introduced at the position of the center of mass of each small molecule of interest on the pore axis of the hexamer (Fig 1C). The resulting free energy landscapes revealed the unbiased probability of finding a nucleotide at each point along the PV; therefore, the energy basins in proximity to Arg18 for dNTPs, rNTPs, NMPs, IP_6_, and BHC were indicative of the preferences for these molecules to bind to that region (Fig 1D, S1 Fig, S1 Table). The free energy of binding for monophosphates was significantly lower compared to triphosphates, indicating that the phosphate groups dominate these dNTP CA interactions. Overall, based on free energies, both IP_6_ and dNTPs resulted in a more stable complex compared to apo CA. Unexpectedly, the depth of the energy basin differed between nucleotide types, as a minimum free energy of −7 to −8 kcal/mol was observed for dATP and dGTP, while a minimum of −10 to −12 kcal/mol was observed for dCTP and dTTP. However, once bound, release of the dNTP required overcoming a barrier of at least 6 kcal/mol; thus, dNTPs translocation through CA hexamers on their own is energetically unfavorable.

**Fig 2:**
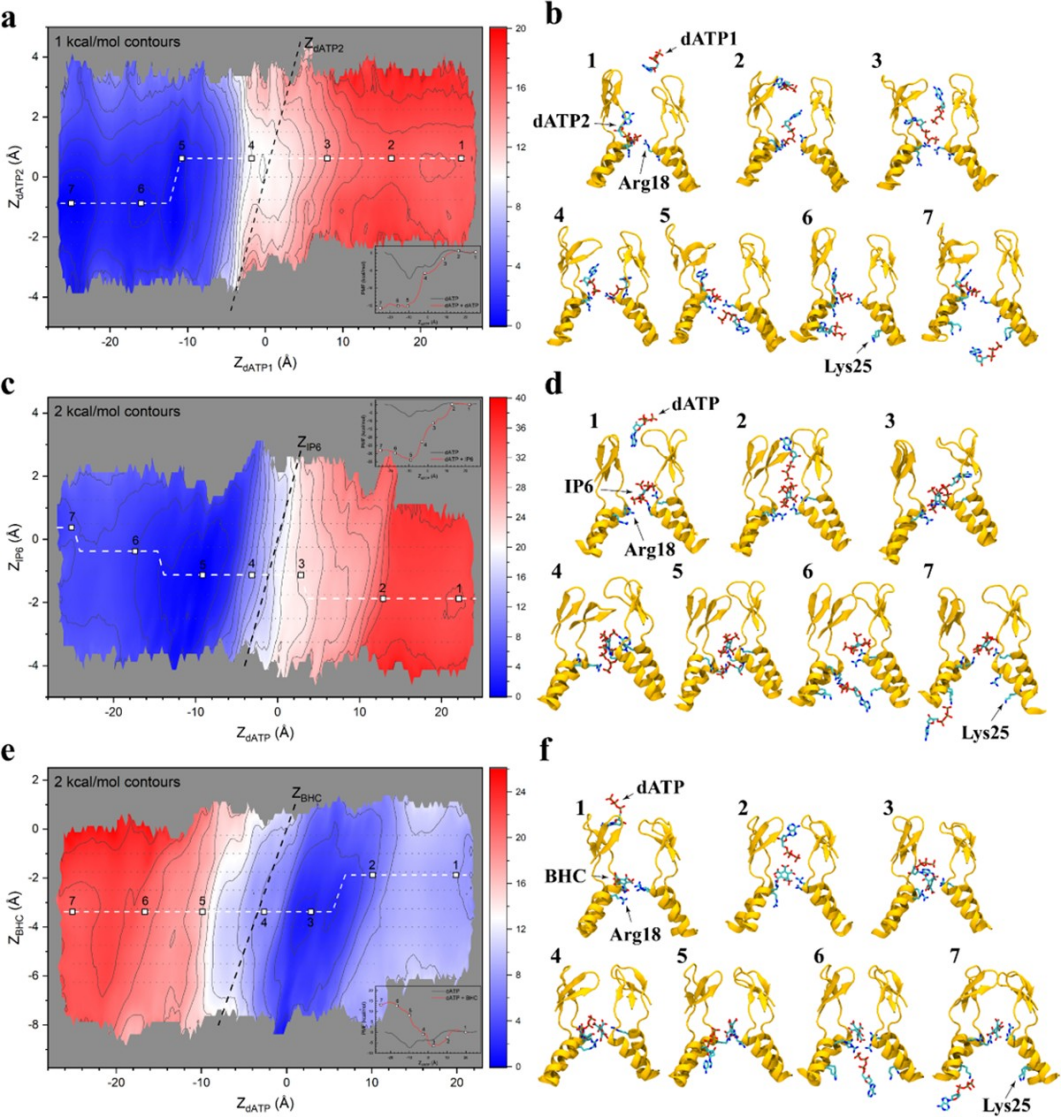
Cooperative binding of small molecules to the central hexamer cavity. Two-dimensional free energy landscapes of dATP translocation through the cavity, in the presence of (a) an additional dATP, (c) IP_6_, or BHC (e). The horizontal reaction coordinates (in Å) represent the location of the COM of a dATP molecule on the cavity axis, which connects the exterior and interior of the capsid (respectively right and left). The vertical reaction coordinates (in Å) are the positions of the COM of (a) a second dATP, (c) IP_6_, or (e) BHC on the progress variable along the central hexamer axis. The magnitudes of the free energies are indicated in the color scalebar in units of kcal/mol. Pathways connecting the interior and exterior are shown as a dashed line, and representative structures corresponding to these translocation events are illustrated in (b), (d), and (f). Numerical data for panels A, C, and E can be found in S2 Data. BHC, benzenehexacarboxylic acid; COM, center of mass; dATP, deoxyadenosine triphosphate; IP_6_, inositol hexakisphosphate.

In our simulations, a single nucleotide tended to reside within the cavity, as the energy well was too deep to enable its release. Our MD simulations revealed that the central hexamer cavity could accommodate 2 small molecules simultaneously to interact with the ring of Arg18 residues (S1 Movie, S2 Fig; molecular simulation #25, 26 in S1 Table). Therefore, we increased the stoichiometric ratio between small molecules and CA hexamer to 2:1 (simulation #11, #12, and #13 in S1 Table; Fig 2, S3 Fig) to examine nucleotide translocation in the presence of multiple charged molecules inside the cavity. Specifically, the free energy profile of a first molecule (shown in the horizontal axes of Fig 2A, 2C and 2E) was calculated throughout the cavity in the presence of a second molecule bound to the capsid cavity near Arg18 (shown in the vertical axes of Fig 2A, 2C and 2E); the closest atomic distances between ligands and Arg18/Lys25 are shown in S4 Fig and S2–S7 Tables. These calculations revealed a second free-energy minimum in the interior of the capsid near Lys25 for a dNTP in the presence of IP_6_ or another dNTP (S31 Movie; Fig 2A and 2C, S3 Fig). Contact analysis between Lys25 and the dNTPs reveals that the former plays a role to coordinate solvent molecules around the nucleotide after dewetting of the dNTP through its binding to Arg18 (S5 Fig). Moreover, the minimum action path connecting the interior and exterior of the capsid (dashed lines in Fig 2A, 2C, and 2E, S3 Fig) demonstrated a marked difference in the ability of CA hexamers to translocate dNTPs in the presence of BHC, IP_6_, or another dNTP. Specifically, a positive free-energy difference, which indicated a high probability of dNTPs to displace inwards, was observed for dNTPs and IP_6_ (Fig 2A and 2C, S3B and S3C Fig), while a negative difference was observed in the presence of BHC (Fig 2E, S3D Fig). The negative difference induced by BHC implied that it inhibits nucleotide import, as the probability of finding a nucleotide below R18 was comparatively low. The origin of the free energy differences between BHC and IP_6_ is related to their mode of binding to the R18 ring. We determined that BHC prefers to lay “flat” on the R18 ring, while IP_6_ tends to stay perpendicular to the ring (S6 Fig). Finally, the free energy difference induced by IP_6_ compared to dNTPs showed that the former is a stronger activator of nucleotide import. Our molecular simulations revealed that IP_6_ will increase dNTP transport, whereas BHC will block or reduce it.

**Fig 3:**
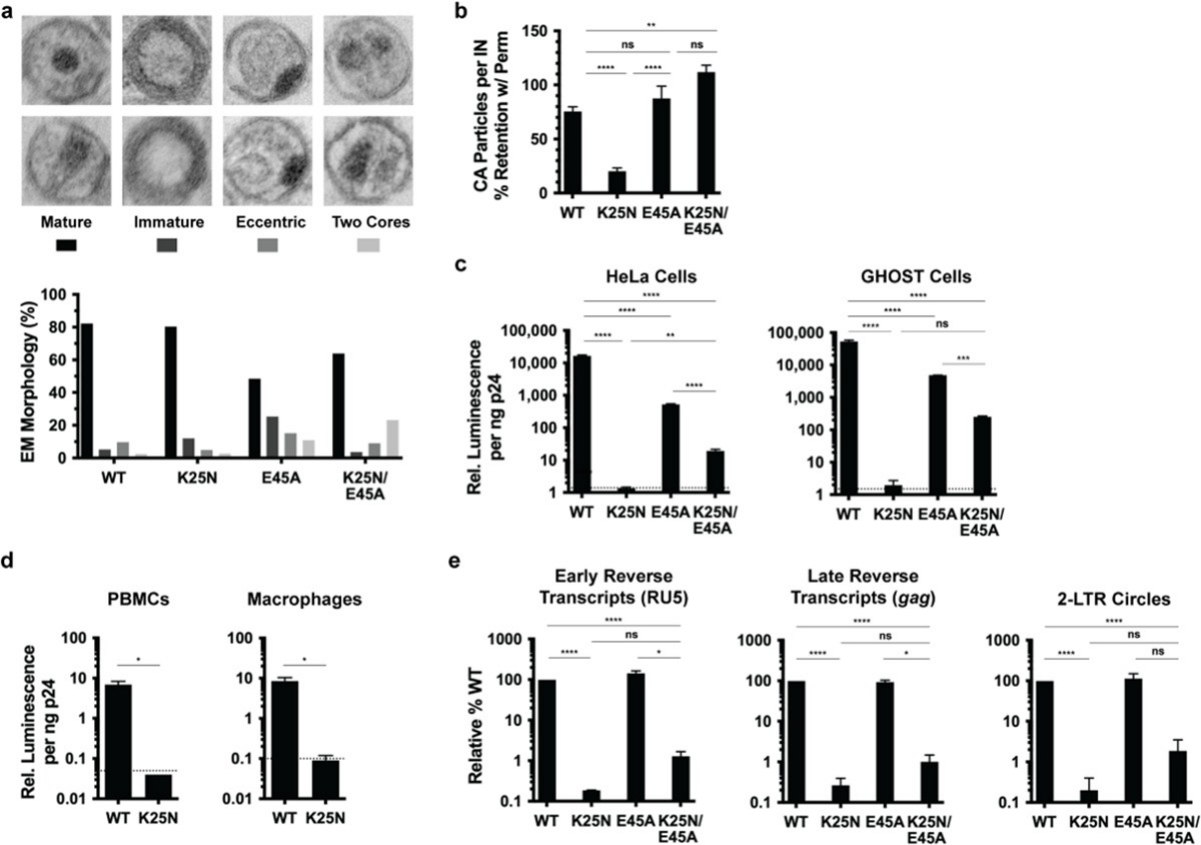
Characterization of K25N HIV-1 and K25N/E45A HIV-1. (a) TEM classification of virus particle morphologies for HIV-1_NL4-3_ bearing indicated CA changes are shown for 2 independent experiments, with >200 particles counted per experiment for each virus type (>480 particles of WT and each mutant virus across experiments). Representative images of the different morphologies are shown for illustrative purposes. Statistical significance between WT and mutant morphologies are indicated. (b) Viruses bearing the indicated CA substitutions were captured on glass with or without virus membrane permeabilization, followed by immunostaining for CA and imaging. Data are expressed as the percentage of non-permeabilized CA staining retained with membrane permeabilization for each virus. (c, d) Single-cycle infectivities of WT and indicated CA mutant viruses in indicated cells. Dotted lines represent average luciferase signal of uninfected cells. (e) DNA synthesis and 2-LTR circle formation of WT and CA mutant viruses. Error bars indicate SEM for 2–4 experiments. **** *P* < 0.0001, *** *P* < 0.001, ** *P* < 0.01, * *P* < 0.05. Numerical data for panels A, B, C, D, and E can be found in S3 Data. 2-LTR, 2-long terminal repeat; TEM, transmission electron microscopy; IN, integrase; ns, not significant; PBMC, peripheral blood mononuclear cell; WT, wild-type.

Results from our simulations showed that Lys25 and Glu28 substitutions modified dNTP import (simulations #14 to 17 in S1 Table and S1D Fig). In particular, K25A and K25N were predicted to inhibit translocation. The prediction of impaired dNTP translocation suggested that reverse transcription should likewise be impaired for K25A and K25N HIV-1, leading to decreased infectivity. We therefore examined Lys25 mutations K25A and K25N in HIV-1 CA. TEM of in vitro assembly reactions revealed that K25A constructs were unable to form mature-like conical capsids (S7 Fig) and therefore were not examined further. K25N constructs, however, were shown by both TEM (S7 Fig) and magic angle spinning (MAS) NMR (S8 Fig) to have similar properties as wild-type (WT) constructs, suggesting that K25N CA should produce virus particles similar to WT HIV-1. To verify this, we produced WT and K25N HIV-1_NL4-3_ viruses, which by TEM showed particles with similar, predominantly mature capsid morphologies (Fig 3A).

Alterations of Lys25 were previously found to produce virus-like particles or viruses with altered capsid stability [21,22]. Approximately half of virion-incorporated CA is incorporated into the capsid structure [23]; the remaining CA complement is comparatively soluble and therefore susceptible to release from virus particles via mild detergent treatment. To examine the stability of K25N HIV-1 capsid, we accordingly employed an imaging-based in vitro CA retention assay whereby viruses containing fluorescently tagged IN (mRuby3-IN) were adhered to glass and briefly permeabilized to allow soluble CA release and retention of intact capsids within the virus membrane (S9A Fig) [24,25]. Samples were fixed, immunostained for CA (p24), and imaged via confocal microscopy. CA staining and mRuby3-IN-containing particles were modeled and enumerated, and the number of stained CA puncta per field was normalized to the total number of mRuby3-IN particles. Retention of a higher number of CA puncta per IN particles is indicative of a more stable capsid, which was confirmed by comparing WT HIV-1 to viruses with previously characterized hyperstable E45A CA and hypostable K203A CA amino acid substitutions [26,27] (S9B and S9C Fig). K25N HIV-1 retained significantly fewer CA puncta compared with WT HIV-1 (Fig 3B), indicating that K25N capsids are hypostable.

To evaluate virus infectivity in cells that naturally produce IP_6_, cell lines, peripheral blood mononuclear cells (PBMCs), and monocyte-derived macrophages were infected with normalized amounts of WT or K25N HIV-1_NL4-3_ that encoded the luciferase reporter gene. While WT HIV-1 infected all cell types tested, K25N was noninfectious (Fig 3C and 3D). To determine the step of the virus life cycle at which K25N HIV-1 infection was impaired, early and late reverse transcripts and 2-long terminal repeat (LTR)-containing circles were measured by quantitative PCR (qPCR). Levels of K25N early and late reverse transcripts were reduced 540-fold and 385-fold, respectively, relative to WT HIV-1 (Fig 3E). The number of 2-LTR circles, a surrogate marker of vDNA nuclear entry, was likewise 500-fold lower for K25N compared to WT HIV-1 (Fig 3E). These data indicate that K25N HIV-1 is defective for reverse transcription.

The instability of K25N HIV-1 capsids could itself result in impaired reverse transcription, obfuscating the potential effects of impaired dNTP import. We hypothesized that stabilized K25N HIV-1 capsids would still be impaired for dNTP import and, thus, attenuated for reverse transcription. In an attempt to stabilize K25N capsids, we incorporated the aforementioned hyperstabilizing CA change E45A [26,27] into K25N HIV-1. Indeed, K25N/E45A HIV-1 revealed significantly greater capsid stability than K25N HIV-1, mimicking the hyperstability phenotype of the E45A capsid (Fig 3B), though both E45A HIV-1 and K25N/E45A HIV-1 produced higher percentages of morphologically defective particles than either WT or K25N HIV-1 (Fig 3A). E45A HIV-1 was 10- to 30-fold less infectious than WT HIV-1 (Fig 3C), consistent with prior characterization of this mutant [26], while K25N/E45A HIV-1 harbored a 200- to 800-fold infectivity defect (Fig 3C). While E45A HIV-1 synthesized WT levels of early and late reverse transcripts and 2-LTR circles (Fig 3E), K25N/E45A HIV-1 early and late reverse transcripts were reduced 78-fold and 100-fold, respectively, and 2-LTR circles were reduced 54-fold (Fig 3E). Thus, the E45A stabilization change can separate the capsid stability and vDNA synthesis phenotypes of K25N HIV-1, suggesting that impaired dNTP import underlies the observed K25N/E45A HIV-1 reverse transcription and infectivity defects. By extension, we infer that impaired dNTP import limits K25N HIV-1 reverse transcription and infectivity.

**Fig 4:**
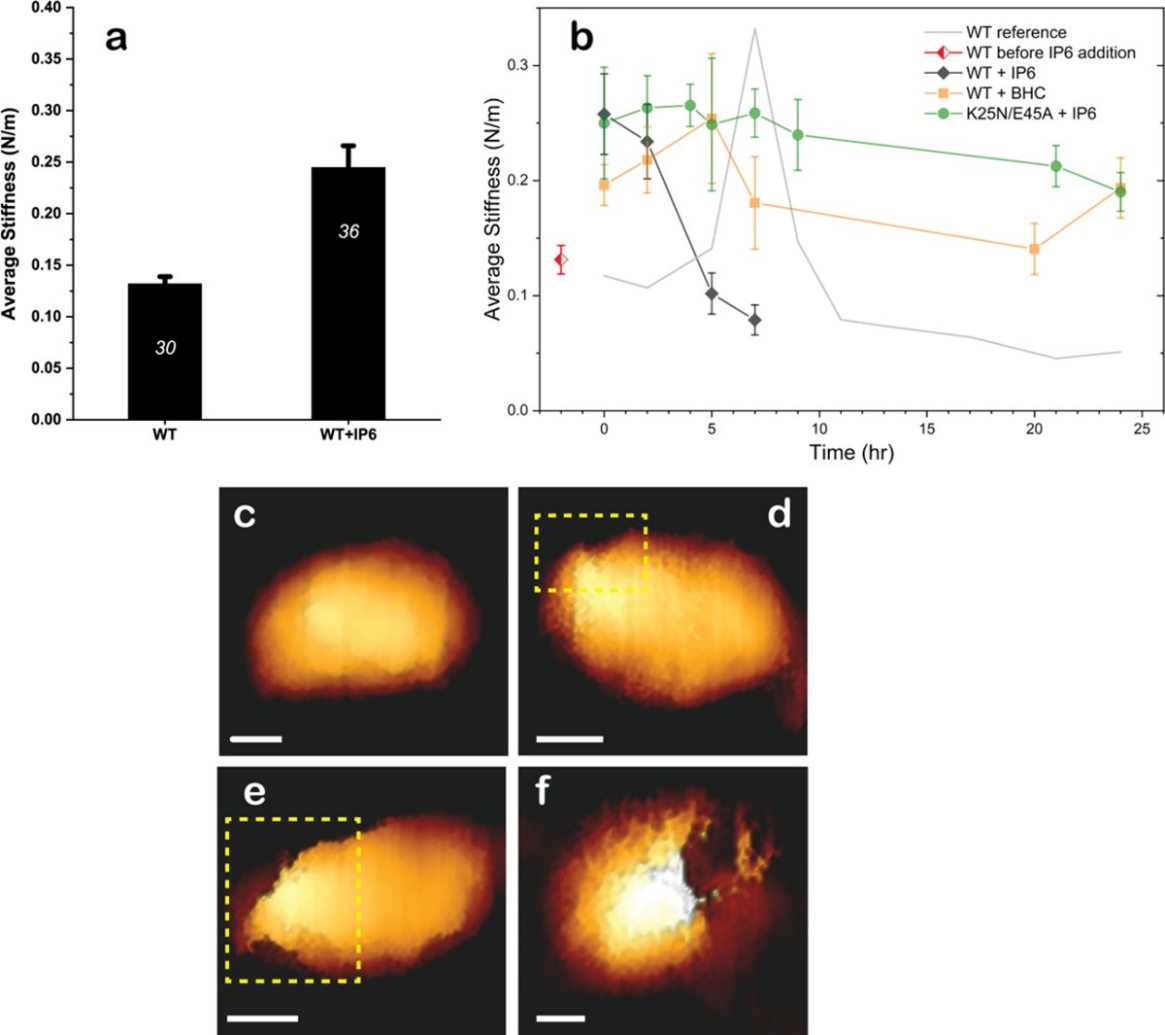
Morphologies and mechanical properties of HIV-1 capsids from AFM. (a) The average stiffness of isolated WT HIV-1 capsids in the absence (*n* = 30) and presence (*n* = 36) of IP_6_. Error bars indicate SEM. Statistical significance was confirmed using the Mann–Whitney U test for *p* < 0.05. (b) Average stiffness of HIV-1 capsids treated with 100 μM IP_6_ (black) or BHC (orange) as a function of time after the addition of dNTPs and MgCl_2_. The red diamond represents the average stiffness of WT capsids before addition of IP_6_ or BHC. The initial average stiffness, at time 0, was measured before addition of dNTPs and MgCl_2_. At each time point, the stiffness of 2 to 4 capsids was measured without stopping the reaction. WT data (taken from [17]) is presented as a gray line for comparison. The error bars represent SEM. (c) A representative cone-shaped capsid (of 20 imaged) prior to addition of dNTP and MgCl_2_. (d–f) Deformed and damaged capsids visualized after 5 h. For clarity, openings in the capsids are shown within a dashed yellow rectangle. Scale bars, 50 nm. A total of 52 capsids were visualized. (Approximately 400 force-distance curves were obtained from individual capsids). Numerical data for panel A and B can be found in S4 Data. AFM, atomic force microscopy; BHC, benzenehexacarboxylic acid; dNTP, deoxynucleotide triphosphate; IP_6_, inositol hexakisphosphate; WT, wild-type.

Changes in capsid stiffness and morphologies during reverse transcription were monitored in real-time by AFM (Fig 4) [17,28,29]. The effect of IP_6_ binding on the stiffness of intact HIV-1 capsids was analyzed using AFM operated in the nanoindentation mode. Isolated WT capsids, in agreement with our previously reported findings, had an average stiffness value of 0.132 ± 0.007 N/m (*n* = 30), and IP_6_ binding increased their stiffness by nearly 2-fold to an average value of 0.245 ± 0.021 N/m (*n* = 36) (Fig 4A). Statistical significance was confirmed using the Mann–Whitney U test for *p* < 0.05. Likewise, in an experimental time course, untreated WT capsids exhibited an average stiffness value of 0.131 ± 0.012 N/m (*n* = 11) and IP_6_ increased this value to 0.258 ± 0.035 N/m (*n* = 15, time = 0 h). However, in contrast to untreated WT capsids, IP_6_-treated capsids progressively lost their stiffness over time, reaching a minimum of 0.078 ± 0.013 N/m (*n* = 7) at 7 h; the minimum stiffness value was reached earlier for capsids treated with IP_6_ than for capsids without IP_6_ (5 h compared to approximately 20 h) [28]. Overall, our AFM measurements showed that addition of IP_6_ initially stiffens the capsids, then induces faster softening after initiation of reverse transcription. In contrast, addition of the synthetic molecule BHC resulted in a smaller initial increase in stiffness (0.196 ± 0.043 N/m; *n* = 4; Fig 4B) which returned to baseline and then remained largely unchanged during the experimental time course. These data are consistent with previous results showing that IP_6_ stabilizes HIV-1 capsids and promotes viral DNA synthesis [12] and our computational experiments that revealed that BHC blocked nucleotide translocation through the CA hexameric cavity.

To further characterize the effects of IP_6_ on reverse transcription, we analyzed the morphologies of IP_6_-treated WT capsids during reverse transcription by AFM operated in the quantitative imaging mode. Prior to reverse transcription, capsids had a well-defined conical appearance. A representative capsid (out of a total of 20 that were imaged) is shown in Fig 4C. At 5 h after adding dNTPs and MgCl_2_ to induce reverse transcription, openings at various sizes in the capsid appeared (Fig 4D–4F). Similar to our previous findings [30,31], the openings were localized exclusively at or near the narrow end of the capsids. Untreated and IP_6_-treated HIV-1 capsids underwent complete disassembly during the time course of the experiments. However, complete disassembly was 3.4 times faster in the presence of IP_6_ (7 h versus to 24 h). Analysis of the reactions beyond 7 h of reverse transcription revealed [32] mostly fragments of various sizes that lacked a defined morphology (from a total of 52 capsids, 11 remained intact). Overall, we found that IP_6_ accelerated capsid disassembly during reverse transcription.

The stiffness of K25N/E45A capsids was also measured during reverse transcription. While the stability of these capsids was insufficient to withstand the full duration of the experiment due to spontaneous disassembly, their stiffness remained unchanged over 5 h (analysis of 32 cores). Although addition of IP_6_ did not affect CA retention of K25N/E45A capsids (S10 Fig), we nonetheless were able to monitor their stiffness values during 24 h of reverse transcription. The stiffness of the IP_6_-treated double mutant capsids (45 cores analyzed) remained unchanged over the experimental time course (Fig 4B), which is consistent with the observed reverse transcription defect of K25N/E45A HIV-1.

**Fig 5:**
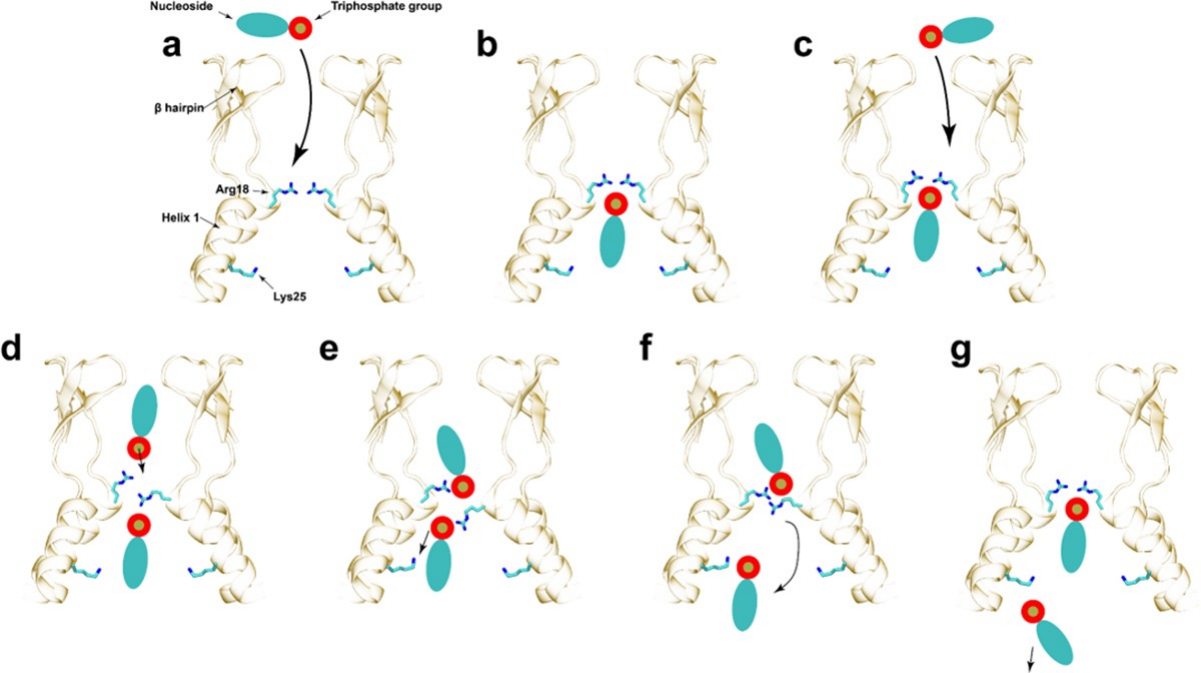
Molecular mechanism for nucleotide translocation through the HIV-1 CA hexamer. (a) A nucleotide diffuses freely between the exterior (top) of the capsid and the central cavity. (b) Subsequently, the nucleotide binds to Arg18 and Lys25 in a canonical binding conformation. Exact interactions between the nucleoside and helix-1 confer nucleotide-type specificity. (c) A second nucleotide freely diffuses between the exterior and the central cavity of CA. (d) The phosphate group of the second nucleotide interacts with Arg18 delocalizing the interactions between the first nucleotide and the Arg18 ring. (e) The second nucleotide enhances interactions between Lys25 and the first nucleotide. (f) Interactions between Lys25 and the phosphate group are weak and thermal fluctuations facilitate dissociation of the dNTP into the interior of the capsid. (g) The second nucleotide now occupies the canonical binding position (b) for a single nucleotide in the cavity.

Reverse transcription and the consequent disassembly of the HIV-1 capsid are fueled by the ability of the capsid to import dNTPs. Free energy calculations revealed a molecular dynamic view of the interaction between a freely diffusing nucleotide and the HIV-1 CA hexamer (Fig 5). First, a nucleotide freely diffused from the exterior solvent to the beta-hairpin region. Subsequently, the loss of entropy by binding of the nucleotide to Arg18 was compensated by the strength of the electrostatic interactions between Arg18 and dNTP phosphate residues. Hydrogen bonds between the nucleotide base and polar residues in helix 1 conferred distinct binding affinities for individual nucleotide species, which acted as a selectivity filter. However, to be released into the capsid lumen, a single nucleotide needs to overcome a high energetic penalty (free energy barrier of over 6 kcal/mol). Engagement of a second charged molecule such as dNTP or IP_6_ within the channel provided the required energy to release the initial dNTP within the capsid lumen.

Hypostable capsid mutants, such as K203A, likely bind IP_6_ but are unstable in cells where IP_6_ concentrations are high. Thus, capsid instability can be independent of IP_6_, which we tested here by combining the hyperstable E45A change with K25N. The lack of IP_6_ stabilization of K25N capsids may be due to a similar general hypostability. Alternatively, the source of K25N hypostability could stem from the interaction between IP_6_ with Lys25 in CA pentamers, thereby reducing the IP_6_ stabilizing effect. To investigate this possibility, we performed MD simulations of IP_6_ bound to CA pentamers, which revealed 100% occupancy of Lys25 by IP_6_ (S11 Fig). The observation that the E45A change, which itself is stabilized by IP_6_, did not induce IP_6_ stabilization of K25N/E45A capsids, lends credence to our finding that IP_6_ stabilizes CA pentamers. Therefore, we conclude that IP_6_ plays a dual role: (1) stabilizing capsid pentamers by interacting with K25; and (2) regulating nucleotide import.

Ion channels and transporters have evolved to employ a cooperative translocation mechanism to displace cargos between distinct environments [33,34]. Our free energy calculations for multiple small molecules suggest that binding of 2 nucleotides within the CA cavity promotes dNTP import. Interestingly, similar to water molecules in aquaporins [34], dNTPs flip as they pass through hexamers. Furthermore, pure electrostatic interactions between CA and dNTPs are insufficient to describe the molecular mechanism of dNTP import, as the loss and gain of entropy associated with dewetting dNTPs and Arg18 is a key contributor to the free energy of binding. As a result, the cooperative mechanism for nucleotide translocation is not universal for any set of negatively charged molecules, as IP_6_ promotes dNTP import while BHC inhibits it.

Recent findings indicate that HIV-1 reverse transcription terminates in the nucleus where dNTP concentrations are comparatively high [32,35,36]. Prior to nuclear import, when dNTP concentrations are low, we conclude that HIV-1 has evolved to import dNTPs into the capsid. Molecular determinants of this import mechanism are encoded in highly conserved residues such as Lys25 to facilitate the rewetting of nucleotide after passage through the Arg18 ring. In addition, we conclude that IP_6_ enhances dNTP import for efficient reverse transcription. The discovery of metabolite-dependent nucleotide-specific import provides a unique target for development of new therapies against HIV-1.

## Methods

### CA pentamer model building

The initial coordinates of the cross-linked pentamer, P17C/T19C and N21C/A22C, model were directly derived from the crystal structure (PDB accession code 3P05), missing loops were added using Modeler [37]. Subsequently, all cysteine residues were mutated back to the wild-type HIV-1_NL4-3_ wild-type sequence, and the complete pentamer was then rigid body docked, using Chimera, into the electron microscopy density of the pentamer obtained from intact particles (EMD accession code EMD-3466). To obtain an atomistic model of the 5MCY pentamer, flexible fitting was performed employing the MDFF method as follows [38]. Ions near the surface of the protein were placed using Cionize [39]. Bulk water and Na/Cl ions were then added using VMD, setting the total concentration of NaCl to 150 mM. Each of the resulting systems contained 100,000 total atoms, including solvent. The systems were equilibrated at 310 K and 1 atm for 5 ns while applying positional restraints on all heavy atoms of the protein. MDFF was then applied for 10 ns with a grid scaling of φ = 0.05 coupled only to backbone heavy atoms; EMD-3466 was used as the target density. Domain restraints were applied to maintain the structural integrity of each individual CA domain in the pentamer. Additional restraints were applied in the form of extra bonds to maintain secondary structure integrity and to prevent transitions of cis/trans bonds and chirality errors [40]. MDFF was performed using NAMD 2.12 [41] with the CHARMM36m force field. Simulated density maps needed for determining cross correlation between model and EM data were generated using VMD [41]. The resulting model was labeled 5MCY, as its backbone resembles the PDB entry 5MCY.

### CA hexamer model building

The initial coordinates of the HIV-1 CA hexamer (S12A and S12B Fig) were generated by applying a 6-fold symmetry operation onto a native full-length HIV-1 CA (PDB accession code 4XFX). The 2 loops between residues 5 to 9 and residues 222 to 231, missing in the original structure, were built using Modeler [30]. Once the hexamer was built, the protonation states of titratable residues, namely histidine, asparagine, lysine, and cysteine, were assigned using PDB2PQR [31] (pH 7.4).

### Molecular mechanics parameterization of NTPs, IP_6_, and BHC

Parameters for the negatively charged small molecules (S13 Fig), except ATP, which has parameters available in the CHARMM general force field, were derived by analogy following the CGENFF protocol [42]. The parameter penalties and charge penalties in each generated parameter files were less than 10 indicating good analogy with the available atom types present in CGENFF. A magnesium ion was added to the triphosphate group present in dNTPs. The coordination number of the magnesium ion with the phosphate group of the dNTP was constrained using *coordnum* in Colvars [43].

### Simulation setup

Small charged molecules such as NTP/IP_6_/BHC were placed in the central cavity of the CA hexamer model. The models were then solvated using the TIP3P water model [44]. Additionally, excess of TIP3P water molecules were deleted to transform the cubic water box into a hexagonal orthorhombic cell of dimension 92.328 Å in the $\hat{x}‐\hat{y}$ plane and 90 Å in the ẑ direction. The length of the system in the ẑ direction provided sufficient solvent padding, greater than 24 Å, to avoid interactions between periodic images. Na and Cl ions were then added to neutralize the system using the CIONIZE plugin in VMD [45], and the bulk salt concentration was set to the physiological concentration of 150 mM, as previously described by the authors [46]. The total number of atoms of the resulting CA hexamer models was 60,000 (S12C and S12D Fig). Solvated WT and K25A, K25N, K25E, and K25E/E28K CAs were derived using the mutator plugin in VMD.

### System minimization and equilibration

The solvated systems were then subjected to minimization in 2 stages, both using the conjugated gradient algorithm with linear searching as implemented in NAMD [39]. Each stage consisted of 10,000 steps of energy minimization. During the first stage, only water molecules and ions were free to move, while the CA protein and NTP/IP_6_/BHC molecules were fixed. In the second stage, the backbone atoms of the CA protein were restrained with a force constant of 10.0 kcal mol^−1^ Å^−2^. Convergence of the minimization procedure was confirmed once the variance of the gradient was below 0.1 kcal mol^−1^ Å^−1^. Following minimization, the systems were tempered from 50 K to 310 K in increments of 20 K over 1 ns. Subsequently, the systems were equilibrated at 310 K for 100,000 steps, while the protein backbone atoms were restrained. Equilibration of the systems was assessed using the RMSD trace versus time plateauing at 2 Å (S14A Fig), the local RMSF per amino acid showing fluctuations less than 1 Å for residues located in helical regions (S14B Fig), and by monitoring the convergence of the radius of the pore in the CA hexamer (S14C Fig). Then positional restraints were gradually released at a rate of 1.0 Kcal mol^−1^ Å^−2^ per 400 ps from 10.0 Kcal mol^−1^ Å^−2^ to 0.0 Kcal mol^−1^ Å^−2^. All MD simulations were performed using NAMD 2.12 with the CHARMM36m force field [39,47,48]. An internal time step of 2 fs was employed in the multistep r-RESPA integrator as implemented in NAMD, bonded interactions were evaluated every 2 fs, and electrostatics were updated every 4 fs. Temperature was held constant at 310 K using a Langevin thermostat with a coupling constant of 0.1 ps^−1^. Pressure was controlled at 1 bar using a Nose-Hoover Langevin piston barostat with period and decay of 40 ps and 10 ps, respectively. The Shake algorithm was employed to constraint vibrations of all hydrogen atoms. Long-range electrostatics was calculated using the particle-mesh-Ewald summation with a grid size of 1 Å and a cutoff for short-range electrostatics interactions of 12 Å.

### Gibbs free energy calculations

Progress variables (PVs), akin to reaction coordinates, for all free-energy molecular dynamics calculations were chosen as the location on the *z* axis of the center of mass of the small-molecule(s) of interest. The origin of the progress variable was set to the center of mass of C_α_ atoms in the N-terminal domain of the CA hexamer (Fig 1C). One- and two-dimensional (2D) potentials of mean force (PMFs) along the PVs were calculated using the Hamiltonian Replica-exchange/Umbrella Sampling (HREX/US) method [43,49,50] implemented in NAMD 2.12 [39,43,51]. The initial coordinates for the HREX/US windows were derived from constant-velocity steered MD (SMD) simulations in which molecules were pulled along the PV at a rate of 0.1 nm/ns. The center of mass of the small-molecules were positionally resta rained in the US windows with a harmonic force constant of 2.5 Kcal mol^−1^ Å^−2^ using Colvars [49]. The width of all US windows was set to 0.75 Å; except for single nucleotide HREX simulations (simulation #1 to 8) in which the window width was set to 1.0 Å. The number of US windows in the present HREX simulations were chosen so that the nucleotide translocation from capsid exterior to interior through the central cavity was uniformly sampled (Fig 1C).

2D HREX/US simulations were employed to study the cooperativity between small molecules for translocation. For this purpose, 2 PVs were employed: PV1 that determined the location of dATP, and PV2 that determined the location of dATP, IP_6_, or BHC in the CA cavity. The initial configurations for the 2D simulations were derived by pulling dATP using constant velocity steered MD [52] at 0.1 nm/ns along PV1, while the center of mass of dATP/IP_6_/BHC was restrained with a harmonic force constant of 2.5 Kcal mol^−1^ Å^−2^ using Colvars (cycle 1, S15A–S15C Fig). A seeding method similar to that reported [53] was employed to generate new simulation windows along PV2 as follows. For each conformation resulting from the SMD simulation, 2 new seeds were generated by displacing by ±0.75 Å the harmonic restraints for dATP/IP_6_/BHC along PV2 (cycle 2, S15A–S15C Fig). Subsequently, 5 ns of HREX/US MD simulation were performed. The last configuration from each replica was then used as the initial seed for a subsequent cycle where the harmonic restraints were displaced by another ±0.75 Å along PV2 (cycle 3, S15A–S15C Fig). The seeding process was repeated one last time and production 2D HREX/US simulations (production runs, S15A–S15C Fig) were performed for 30 ns per window. During each HREX/US simulation, exchange of the harmonic potential between neighboring replicas was attempted every 1000 steps (2 ps). Exchanges were attempted randomly between successive replicas; trial attempts for all replicas were equally probable. The following Metropolis Monte Carlo exchange criterion [50] was employed

$$p_{exchange}=min(1,e^{-\frac{{[U}_{i}(q_{i})-U_{i}(q_{j})]+[U_{j}(q_{j})-U_{j}(q_{i})]}{k_{B}T}})$$

where T = 310 K, *k_B_* is the Boltzmann constant, *q_i_* and *q_j_* denote the 3D conformations for 2 replicas i and j, and *U_i_* and *U_j_* represent the potential energies derived from the restrained Hamiltonian evaluated at the indicated conformation.

Potentials of mean force were derived from the resulting sampling in each of the US windows using the weighed histogram analysis method (WHAM) [53,54]. In WHAM, PMF bins are obtained from US/HREX simulation windows. Convergence of the 1D US/HREX calculations was characterized by the changes in the resulting PMF in trajectory increments of 10 ns (S16 Fig). That is, simulations were considered to have converged once the maximum change in one of the PMF bins resulting from adding more simulation data was less than 1 Kcal mol^−1^.

Convergence of the 2D calculations was examined by means of the fluctuations of the average root mean squared error for each PMF bin (RMSE) (S17A Fig). Each 2D HREX/US simulation was divided into 60 time-slices of width 0.5 ns. Then, the PMF was calculated for each slice using WHAM. Using the last slice as the reference, the RMSE of the *k-*th slice was calculated using the equation:

$$RMSE(k)=\sqrt{\frac{\sum_{i=1}^{N_{bins}}{\left(G_{i,k}-G_{i,reference}\right)}^{2}}{N_{bins}}}$$

where the sum runs over the total number of PMF bins (*N*_*bins*_), G_*i*, *k*_ and G_*i*, *reference*_ are the free energies at the *i*-th PMF bin for the *k*-th and last slice, respectively. Convergence of 2D PMF was established once the RMSE was not larger than 3.0 kcal/mol. The bins before convergence were discarded. Subsequently, utilizing the converged slices, the 2D PMF surfaces (Fig 2) and their standard deviations (S17B–S17D Fig) were computed using WHAM. The statistics of the exchange ratios between neighboring US replicas in HREX simulations are shown in S18 Fig. Overall, the exchange ratios are greater than 20% in 2D HREX simulations, indicating enough sampling efficiencies (S18 Fig).

### Cell lines

HEK 293T, GHOST, and HeLa cells were maintained at 37°C in Dulbecco's modified Eagle medium supplemented with 10% heat-inactivated bovine serum, 1% penicillin-streptomycin, and 1% glutamine in 5% CO_2_. Human PBMCs were isolated from leukopheresis packs obtained from the Central Blood Bank (Pittsburgh, Pennsylvania) via Ficoll-Paque Plus (GE Healthcare, Marlborough, Massachusetts) density gradient centrifugation, following manufacturer’s instructions, and stimulated with 50 U/mL recombinant interleukin-2 (IL-2, Thermo Fisher, Waltham, Massachusetts) and 5 μg/mL phytohaemagglutinin (PHA, Thermo Fisher) for 48 to 72 h prior to infection. CD14+ monocytes were purified from PBMCs via CD14 MicroBeads (Miltenyi, Auburn, California) magnetic cell sorting, following manufacturer’s instructions, and differentiated into macrophages with 50 ng/mL granulocyte macrophage colony-stimulating factor (GM-CSF, R&D Systems, Minneapolis, Minnesota) for 6 d prior to infection.

### Isolation of HIV-1 capsids for AFM measurements

HIV-1 pseudovirions were used to isolate capsids as previously described [55,56]. Briefly, approximately 10^6^ human embryonic kidney (HEK) 293T cells were transfected with 2.5 μg of ΔEnv IN- HIV-1 plasmid (DHIV3-GFP-D116G) [17] using 10 μg of polyethylenimine (PEI, branched, MW approximately 25,000, Sigma-Aldrich, St. Louis, Missouri). After 20 h, the medium was replaced with fresh medium. After 6 h, the supernatant was harvested, centrifuged at 1,000 rpm for 10 min, and filtered through a 0.45-μm-pore size filter. The virus-containing supernatant was concentrated by ultracentrifugation in an SW-28 rotor (25,000 rpm, 2 h, 4°C) using OptiPrep density gradient medium (Sigma-Aldrich, Darmstadt, Germany). Virus containing fractions were collected, mixed with 10 ml of TNE buffer (50 mM Tris-HCl, 100 mM NaCl, 0.1 mM EDTA (pH 7.4)) and added to 100-kDa molecular mass cutoff Vivaspin 20 centrifugal concentrators (100,000 MWCO, Sartorius AG, Germany). The mixture was centrifuged twice at 2,500 × g for 25 to 30 min at 4°C, until the supernatant level in the concentrators reached 200 to 300 μL.

Capsids were isolated from concentrated virus-containing material using a previously described protocol [57] with modifications. Briefly, approximately 40 μL of purified HIV-1 pseudovirions was mixed with an equal amount of 1% Triton-X diluted in 100 mM 3-(N-morpholino) propane sulfonic acid MOPS buffer (pH 7.0) and incubated for 2 min at 4°C. The mixture was centrifuged at 13,800 × g for 8 min at 4°C. After removing the supernatant, the pellet was washed twice by adding approximately 80 μl of MOPS buffer and centrifuging at 13,800 × g for 8 min at 4°C. The pellet was resuspended in 10 μl of MOPS buffer.

### AFM measurements and analysis

AFM measurements and analysis were performed as previously described[17,28]. Briefly, 10 μL of isolated HIV-1 capsids resuspended in MOPS buffer was incubated for 30 min at room temperature on hexamethyldisilazane (HMDS)-coated microscope glass slides. Measurements were carried out in MOPS buffer, without sample fixation. Every experiment was repeated at least 3 times, each time with independently purified HIV-1 capsids. IP_6_ was provided from the James laboratory as a generous gift. Measurements were carried out with a JPK Nanowizard Ultra-Speed atomic force microscope (JPK Instruments, Berlin, Germany) mounted on an inverted optical microscope (Axio Observer; Carl Zeiss, Heidelberg, Germany) using silicon nitride probes (mean cantilever spring constant, kcant = 0.12 N/m, DNP, Bruker, Camarillo, California). Height topographic and mechanical map images were acquired in quantitative imaging (QI) mode, at a rate of 0.5 lines/s and a loading force of 300 pN. All measurements were carried out in MOPS buffer which contained 100 μM IP_6_ or mellitic acid (Sigma) when relevant.

Capsid stiffness was obtained by the nanoindentation method as previously described [17,28,57]. Briefly, the stiffness value of each capsid was determined by acquiring approximately 400 force-distance (F-D) curves. To determine the stiffness value of capsids, 20 F-D curves at a rate of 20 Hz at each of 24 different points on the capsid surface were determined. To confirm that the capsid remained stable during the entire indentation experiment, we monitored individual measured point stiffness as a histogram (S19A Fig) and as a function of the measurement count (S19B Fig). Samples whose point stiffness values decreased consistently during experimentation were discarded, since they underwent irreversible deformation.

The maximum indentation of the sample was 4 nm, which corresponds to a maximum loading force of 0.2 to 1.5 nN. Prior to analysis, each curve was shifted to set the deflection in the noncontact section to zero. The set of force distance curves was then averaged (S19C Fig). From the slope of the averaged F-D curve, measured stiffness was derived mathematically. The stiffness of the capsid was computed using Hooke's law on the assumption that the experimental system may be modeled as 2 springs (the capsid and the cantilever) arranged in series. The spring constant of the cantilever was determined during experiment by measuring thermal fluctuation [58]. To reduce the error in the calculated point stiffness, we chose cantilevers such that the measured point stiffness was <70% of the cantilever spring constant. Data analysis was carried out using MATLAB software (The Math Works, Natick, Massachusetts).

### Viruses

Mutations were generated in previously described single-round plasmid derivatives of HIV-1_NL4-3_ (pNLdE-luc) [59] that encode for luciferase via the QuikChange site-directed PCR mutagenesis kit (Agilent, Santa Clara, California). Resulting plasmid DNAs were verified by Sanger sequencing. Virus was produced in HEK 293T cells via transfection of the proviral plasmids with pL-VSV-G [60] and, for imaging assays, pcDNA5-TO-Vpr-mRuby3-IN [61] using Lipofectamine 2000 (Thermo Fisher). Supernatants were harvested after 48 h, centrifuged to remove cells, and stored in aliquots at −80°C. The infectivity of HIV-1 stocks normalized by p24 content (XpressBio, Frederick, Maryland) was assayed by infection of GHOST cells as previously described [62].

### ERT with WT and CA-mutant protein for AFM analysis

Reverse transcription was induced in isolated capsids attached to HMDS-coated microscope glass slides. To initiate reverse transcription, MOPS buffer was replaced with reverse transcription buffer (100 μM dNTPs and 1 mM MgCl_2_ in 100 mM MOPS buffer (pH 7.0)) [29]. All measurements were carried out at room temperature (23 to 25°C).

### HIV-1 CA retention assay

Equal p24 amounts of virus containing mRuby3-IN packaged in trans were diluted in STE buffer (100 mM NaCl, 10 mM Tris-HCl, 1 mM EDTA (pH 7.4)) supplemented with 100 μM IP_6_ and adhered to 35 mm glass-bottom dishes (MatTek, Ashland, Massachusetts) coated with Cell-Tak (Corning) for 30 min at 37°C following manufacturer’s instructions. Samples were washed with PBS and briefly permeabilized with 0.02% Triton X-100 (Thermo Fisher, Waltham, Massachusetts) for 30 s, then again washed with PBS and fixed with 2% paraformaldehyde (PFA). For IP_6_ studies, samples that had been adhered to glass and briefly permeabilized as above were additionally incubated for 2 h at 37°C in STE buffer with or without 100 μM IP_6_. To address the possibility that the preincubation brief permeabilization was transient and the virus membrane had resealed during the 2-h incubation (thereby trapping loose CA within the virus membrane), samples were briefly permeabilized again after incubation to ensure loose CA was able to disperse, prior to washing and fixation. Fixed samples were permeabilized with 0.1% Triton X-100, blocked with normal donkey serum, immunostained for CA/p24 using mouse anti-CA monoclonal antibody 24–4 (Santa Cruz, Dallas, Texas) and donkey anti-mouse IgG-Cy5, and mounted with coverslips. Confocal imaging was performed on a Nikon A1 laser scanning confocal microscope, with 6 fields imaged per sample. CA staining and mRuby3-IN-containing particles were modeled and enumerated with Imaris imaging analysis software (Bitplane, Zurich, Switzerland). The number of stained CA puncta per field was normalized to the number of mRuby3-IN particles, which were taken as representing intact capsids. The normalized number of CA puncta was reported for each condition (with or without permeabilization) or reported as the percentage of non-permeabilized puncta retained upon permeabilization.

### Infectivity assays and measurement of reverse transcripts and 2-LTR circles

Cells were plated overnight in 24-well or 96-well plates and challenged with virus at equal amounts of p24. Virus infectivity was determined by luciferase production (Promega) after 48 h using a Synergy2 Multi-Detection Microplate Reader (BioTek, Winooski, Vermont)

HeLa cells were plated in 6-well plates and infected with virus at equal amounts of p24 after treatment with DNaseI (Roche, Indianapolis, Indiana) for 30 min at 37°C. Control infections were performed in the presence of 25 nM rilpivirine. After 24 h, cells were washed with PBS, trypsinized, and pelleted, and DNA was extracted using the Blood Mini Kit (Qiagen, Germantown, Maryland). Early (RU5) and late (gag) HIV-1 reverse transcripts and 2-LTR circles were measured by qPCR as previously described [16]. Values obtained in the presence of rilpivirine were subtracted from experimental values to determine levels of viral reverse transcription and 2-LTR circle formation.

### Statistics of infectivity assays, quantitative PCR, and capsid stability assay

Results were analyzed for statistical significance by 2-sided Student *t* test (infectivity assays and quantitative PCR) or 1-way ANOVA with Tukey multiple comparisons test (capsid stability assay) with Prism software (GraphPad, San Diego, California). A *p*-value of less than or equal to 0.05 was used to indicate statistical significance.

### TEM of viruses

Virus produced from HEK 293T cells was centrifuged to remove cells, filtered through a 0.45 μm Polyethersulfone (PES) syringe filter (Millipore, Burlington, Massachusetts), transferred into 25 × 89 mm polyallomer ultracentrifuge tubes (Beckman Coulter, Brea, California), and ultracentrifuged in an Optima XL-100K ultracentrifuge (Beckman Coulter) using an SW28 rotor at 25,500 rpm (117,250 × g) for 2.5 h at 4°C. Following aspiration of supernatant, the pellet was fixed in FGP fixative (1.25% formaldehyde, 2.5% glutaraldehyde, and 0.03% picric acid in 0.1 M sodium cacodylate buffer (pH 7.4)) for 2 h at room temperature and stored at 4°C. Ultrathin sections (60 nm) were cut on a Reichert Ultracut-S microtome, transferred to copper grids stained with lead citrate, and observed using a JEOL 1200EX microscope with an AMT 2k charge-coupled-device camera. Images captured at 30,000× magnification were visually inspected to classify viral particles as mature, immature, eccentric, or containing 2 apparent capsids, and over 100 particles were counted per virus preparation; representative TEM images are shown in S20 and S21 Figs and summarized in S8 Table. Statistical significance versus matched WT morphology was assessed using 2-way ANOVA with Sidak multiple comparisons test with GraphPad Prism software.

### Preparation of recombinant CA assemblies

U-^13^C,^15^N labeled CA K25N and K25A mutants were isolated and purified using the protocol reported previously with minor modifications [16]. Pure proteins were assembled as reported previously [17] with minor modifications. First, the proteins were dialyzed overnight against 50 mM MES buffer (pH 6.0) containing 300 mM NaCl. The dialyzed proteins were concentrated to 40 mg/mL and diluted to 1:1 volumetric ratio with assembly buffer (50 mM MES (pH 6.0), 600 μM NaCl). The final assembly was carried at room temperature by 1:1 volumetric dilution with 1,800 μM inositol hexaphosphate (IP_6_) (pH 6.0). CA assemblies were pelleted after the incubation for 1 h and stored at 4°C.

### Preparation of recombinant CA-SP1-NC assemblies

Protein was purified and assembled as previously described by the authors [57].

### Transmission electron microscopy of CA assemblies

After assembly, a small aliquot of each sample was removed and immediately stained with 2% uranyl acetate on copper grids. Images were collected using the TALOS F200C at the Keck Center for Advanced Microscopy and Microanalysis of Interdisciplinary Science and Engineering (ISE) Laboratory of the University of Delaware.

### MAS NMR Spectroscopy of CA assemblies

MAS NMR experiments were performed on a Bruker 20.0 T narrow bore AVIII spectrometer. The Larmor frequencies of ^1^H, ^13^C, and ^15^N were 850.4, 213.8, and 86.2 MHz, respectively.

The U-^13^C, ^15^N-CA WT and K25A conical assemblies were packed into 3.2 mm thin wall MAS NMR rotors, and ^13^C-^13^C CORD data sets were acquired using a 3.2 mm E-Free HCN probe. The sample temperature was 5 ± 1°C controlled by the Bruker VT controller. The MAS frequency was 14.000 ± 0.001 kHz controlled by the Bruker MAS-3 controller. The 90° pulse lengths were 2.9 μs (^1^H) and 4.3 μs (^13^C). The cross-polarization contact time was 1 ms; a linear 90% to 110% amplitude ramp of was applied on ^1^H; the center of the ramp was at 82 kHz and Hartmann-Hahn matched to the first spinning sideband; ω_RF_ = 54 kHz was applied on the ^13^C channel. SPINAL-64 ^1^H decoupling [63] (ω_RF_ = 86 kHz) was applied during t_1_ and t_2_ periods. The CORD mixing time was 50 ms. The spectra were acquired as a 2,048 × 864 (t_2_ × t_1_) points complex matrix to the final acquisition times of 15.98 and 10.29 ms, respectively. States-TPPI protocol [64] was used for frequency discrimination in the indirect dimension. A total of 156 transients were added up for each FID; the recycle delay was 2 s.

The U-^13^C, ^15^N-CA K25N conical assemblies were packed into a 1.3 mm MAS NMR rotor, and ^13^C-^13^C RFDR spectrum was acquired using a 1.3 mm HCN probe. The sample temperature was 5 ± 1°C controlled by the Bruker VT controller. The MAS frequency was 60.000 ± 0.001 kHz controlled by the Bruker MAS-3 controller. The 90° pulse lengths were 1.4 μs (^1^H) and 2.85 μs (^13^C), with T_CP_ of 1 ms; a linear 90% to 110% amplitude ramp of was applied on ^1^H; the center of the ramp was at 100 kHz and Hartmann-Hahn matched to the first spinning sideband; ω_RF_ = 20 kHz was applied on the ^13^C channel. TPPM ^1^H decoupling [65] (ω_RF_ = 10 kHz) was applied during t_1_ and t_2_ periods. The spectrum was acquired as a 2,048 × 800 (t_2_ × t_1_) points complex matrix to the final acquisition times of 15.98 and 9.84 ms, respectively. States-TPPI was used for frequency discrimination in the indirect dimension. A total of 256 transients were added up for each FID; the recycle delay was 2 s.

The spectra were processed with 50° shifted squared sine-bells in both dimensions and zero-filled to at least twice the number of points in the indirect dimension. The processed spectra were analyzed in NMRFAM-Sparky [66]. Chemical shifts of CA NL_4-3_ tubular assemblies [67,68] were used for data analysis (BMRB: 30741; PDBID: 6X63).

## Supporting information

S1 Fig The 1D free energy landscapes of small molecules interacting with CA hexamers (PDBID: 4XFX). (A) Binding profiles of rNTPs show different binding affinities and specifics compared to dNTPs. (B) Binding profiles of nucleotide monophosphates, which are weaker compared to rNTPs and dNTPs. (C) Binding profile of BHC and IP_6_ to a hexamer. BHC presents a much wider well compared to IP_6_. (D) 1D free energy landscapes of dATP binding to WT CA and indicated mutants. Compared to WT, K25A should result in leaky capsids, in contrast to K25N, K25E, and K25E/E28K, which each should result in blocked capsid cavities. Numerical data for panels A, B, C, and D can be found in S5 Data. BHC, benzenehexacarboxylic acid; dATP, deoxyadenosine triphosphate; dNTP, deoxynucleotide triphosphate; IP_6_, inositol hexakisphosphate; rNTP, ribonucleoside triphosphate; WT, wild-type.
(TIF)

S2 Fig The central CA hexamer cavity can accommodate 2 small molecules (PDBID: 4XFX). The radius of the spheres represent the van der Waals radii for each nuclei, hydrogens are shown in white, phosphates are shown in yellow, oxygen in red, nitrogen in dark blue, and carbon in cyan. The NTD domain of CA is shown in yellow, illustrating helix 1 and the beta-hairpin region. (A) Two dATP molecules near R18 are shown. (B) Two dATP molecules one above R18 and the second below, interacting with K25 are shown. dATP, deoxyadenosine triphosphate; NTD, N terminal domain.
(TIF)

S3 Fig Free energy landscape of cooperative interactions between small molecules and the CA hexamer. (A) Free energy profile for a single dATP interacting with CA. The profile indicates dNTP binding, but not translocation as the energy barrier is too high to be overcome by simple thermal fluctuations. (B) Cooperation between multiple dATP molecules shifts the free energy profile from binding to a gradient pointing toward the interior of the capsid. (C) Cooperation between IP_6_ and dNTPs also creates a gradient toward the interior, but almost twice as strong compared to dNTPs alone. (D) BHC inside of the CA cavity facilitates dNTP binding but not translocation. Numerical data for panels A, B, C and D can be found in S6 Data. BHC, benzenehexacarboxylic acid; dATP, deoxyadenosine triphosphate; dNTP, deoxynucleotide triphosphate; IP_6_, inositol hexakisphosphate.
(TIF)

S4 Fig Closest interligand distances observed in the present study. Numerical data for the plot can be found in S7 Data.
(TIF)

S5 Fig Mean number of water molecules interacting with dNTPs. (A) A significant dewetting of dNTPs is observed near Arg18, indicating a loss of conformational entropy. (B) Similarly, loss of water molecules is observed for rNTPs. The loss of entropy is compensated by the formation of salt-bridges between Arg18 and dNTPs (C) and rNTPs (D). (E) Correlation analysis between loss of solvation molecules and formation of bonds with Arg18 and NTPs. Importantly, solvation of the dNTP/rNTP as it moves toward the interior of the capsid is assisted by several charged or polar residues including K25/30 and E28/29. Numerical data for panels A, B, C, D, and E can be found in S8 Data. dNTP, deoxynucleotide triphosphate; NTP, nucleoside triphosphate; rNTP, ribonucleoside triphosphate.
(TIF)

S6 Fig IP6 and BHC exhibit distinct interactions with CA hexamers. (A) Molecular architecture of the R18 ring in hexamers as observed in the crystal structure (PDBID: 4xfx). (B) Molecular structure of BHC and myo-IP6. (C) Binding of IP_6_ and (D) BHC to the ring of R18. (E) The angle of inclination is defined as the angle between the plane defined by the 6-membered rings and the plane of the CA-hexamer. Time evolution of the inclination angle for IP_6_ and BHC. The 2 molecules exhibit different inclination angles. (F) NCFGs within 3.4 Å of R18. (G) Number of R18 within 3.4 Å of IP_6_ and BHC. Numerical data for panels E, F, and G can be found in S9 Data. BHC, benzenehexacarboxylic acid; IP_6_, inositol hexakisphosphate; NCFG, number of negatively charged functional group.
(TIF)

S7 Fig In vitro assembly of CA-SP1-NC and CA, NL4-3 strains. (A–F) Negatively stained TEM images of WT (A, B), K25N (C, D) and K25A (E, F) CA-SP1-NC. The mutants produce virus-like particles with immature morphologies. Assemblies in (A, C, E) contain 2 mg/ml of the respective protein in 50 mM Tris-HCl (pH 8.0), 100 mM NaCl. Assemblies in (B, D, F) contain 10 mg/ml of the respective protein in 50 mM MES (pH 6.0), 10 μM IP_6_. (G–J) Negatively stained TEM images of WT (G, H) K25N (I) and K25N (J) CA show virus-like particles of mature morphology, namely tubes and cones. Assemblies in panel (G) contain 20 mg/ml CA in 50 mM Tris-HCl (pH 8.0), 2.4 M NaCl. Assemblies in panels H, L, and J contain 20 mg/ml of the respective protein in 50 mM MES (pH 6.0), 9 μM IP_6_. TEM, transmission electron microscopy; WT, wild-type.
(TIF)

S8 Fig 2D 13C-13C correlation MAS NMR spectra of conical assemblies of CA (CORD, magenta) and CA K25N mutant (RFDR, light blue). The assemblies contain 20 mg/ml of the respective protein in 50 mM MES (pH 6.0), 0.9 μM IP6. The similar chemical shifts indicate that K25N mutant is folded and its overall structure is the same as in the WT CA conical assemblies. CORD, combined R2vn-driven spin diffusion; IP6, inositol hexakisphosphate; MAS, magic angle spinning; RFDR, homonuclear radio-frequency driven recoupling; WT, wild-type.
(TIF)

S9 Fig CA retention imaging assay distinguishes WT, hyperstable, and hypostable HIV-1 capsid phenotypes. (A) Schematic of the in vitro capsid stability assay. An intact virus membrane (without permeabilization, left) traps loose CA, such that a stable and unstable capsid have similar stained CA fluorescence signals. Upon light permeabilization of the virus membrane (right), loose CA can diffuse from the virus particle and does not contribute to the stained CA fluorescence signal. Fluorescently labeled IN associated with the RNA genome remains trapped within the lightly permeabilized virus membrane. Comparing the IN-normalized CA staining retention upon light permeabilization permits differentiation of stable and unstable capsid. (B) Viruses packaging mRuby3-IN in trans with WT CA or CA bearing the indicated mutations were captured on glass either with or without prefixation virus membrane permeabilization, immunostained for CA, and imaged. The number of CA particles per IN particles is shown for each virus +/− permeabilization. E45A and K203A HIV-1 are included as examples of hyperstable and hypostable CA mutants, respectively. Error bars indicate SEM for 2 experiments. (C) The mean fluorescence intensity of CA staining for each imaged virus particle is shown, with the overall mean intensity for the population indicated by the purple bar. Representative results are shown from 1 experiment. ** *P* < 0.01; *** *P* < 0.001; **** *P* < 0.0001. Numerical data for panels A and B can be found S10 Data. IN, integrase; WT, wild-type.
(TIF)

S10 Fig The effect of IP6 on HIV-1 CA retention. WT or mutant viruses containing mRuby3-IN were captured on glass, lightly permeabilized, and incubated for 2 h in STE buffer with or without 100 μM IP_6_. Viruses were lightly permeabilized again, fixed, immunostained for CA, and imaged. (A) The number of CA particles per IN particles is shown for each virus +/− IP_6_. Error bars indicate SEM for 2 experiments. (B) The data from panel A are shown expressed as the fold change of CA staining retained for each virus with the addition of IP_6_ during incubation. (C) The mean fluorescence intensity of CA staining for each imaged virus particle is shown, with the overall mean intensity for the population indicated by the purple bar. Representative results are shown from 1 experiment. * *P* < 0.05; ** *P* < 0.01; *** *P* < 0.001; **** *P* < 0.0001. Numerical data for panels A, B, and C can be found in S11 Data. IN, integrase; IP_6_, inositol hexakisphosphate; WT, wild-type.
(TIF)

S11 Fig Binding of IP6 to CA pentamers, namely PDBIDs 5MCY and 3P05. (A) Models based on the crystallographic cross-linked pentamer, and the cryoEM-derived model from intact HIV-1 particles. The figures illustrate the structures of IP6-bound CA pentamers before and after MD simulations. (B) Contact occupancy between CA pentamer residues and IP6. (C) Traces of the root mean squared deviations for alpha carbons in the CA pentamer models. The low RMSD illustrate that IP6 stabilizes both structures. Numerical data for panel C can be found in S12 Data. cryoEM, cryogenic electron microscopy; IP6, inositol hexakisphosphate; MD, molecular dynamics; RMSD, root-mean-square deviation.
(TIF)

S12 Fig Atomistic models employed in the present study based of PDBID: 4XFX. (A) The structure of an HIV-1 CA monomer and (B) hexamer. (C) The CA hexamer in a hexagonal water box with 150 mM NaCl and dATP. (D) A flat CA hexamer lattice formed by applying periodic boundary conditions.
(TIF)

S13 Fig Molecular structures of small molecules used in the present study. dNTP, rNTP, myo-IP_6_, BHC, and dNTP and rNTP bases. BHC, benzenehexacarboxylic acid; dNTP, deoxynucleotide triphosphate; rNTP, ribonucleoside triphosphate.
(TIF)

S14 Fig Convergence of MD simulations employed in the present study. (A) RMSD trace as a function of time during the equilibration phase of the systems. (B) RMSF of CA monomers during equilibration of the systems. (C) Convergence of the radius of the CA hexamers pores. Numerical data for panels A, B, and C can be found in the file S13 Data. MD, molecular dynamics; RMSD, root-mean-square deviation; RMSF, root-mean-square fluctuations.
(TIF)

S15 Fig Setup of the 2D HREX/US simulations. (A) Generation of initial seeds for 2D HREX/US simulations of the 2 dATP models, (B) a dATP with IP_6_ and (C) BHC model, through 3 cycles of 5 ns HREX/US simulations. In each cycle, new US windows were generated to increase the sampling in 2D space. The initial conformations of the first cycles were extracted from SMD simulations pulling dATP through the hexamer central pore. After that, the initial conformations for new US windows (shown as yellow dots) were copied from the last frame of nearest previous US windows (shown as white dots). After 3 cycles, the generated seeds were used in 30 ns production runs. Numerical data for panel A, B and C can be found in the file S14 Data. BHC, benzenehexacarboxylic acid; dATP, deoxyadenosine triphosphate; HREX/US, Hamiltonian Replica-exchange/Umbrella Sampling; IP_6_, inositol hexakisphosphate; SMD, steered MD.
(TIF)

S16 Fig Convergence of the 1D HREX/US simulations. Sequential changes of PMF from every 10 ns HREX/US simulations are shown. After 10 ns, the PMF profiles were used to compute the 1D PMF for NTP translocation. Numerical data for all panels can be found in the file S15 Data. HREX/US, Hamiltonian Replica-exchange/Umbrella Sampling; NTP, nucleoside triphosphate; PMF, potential of mean force.
(TIF)

S17 Fig Convergence of the 2D HREX/US simulations. (A) Root mean squared errors of three 2D HREX/US simulations with 0.5 ns. Standard deviations of the 2D PMF surface of panel (B) the 2 dATP models, (C) dATP with IP_6_ and (D) dATP with BHC. Numerical data for panels A, B, C, and D can be found in the file S16 Data. BHC, benzenehexacarboxylic acid; dATP, deoxyadenosine triphosphate; HREX/US, Hamiltonian Replica-exchange/Umbrella Sampling; IP_6_, inositol hexakisphosphate; PMF, potential of mean force.
(TIF)

S18 Fig Exchange ratios for the HREX/US simulations performed in the present study. (A) 1D rNTP translocation simulation, (B) 1D IP_6_ and BHC translocation simulations, and (C) the three 2D dATP translocation simulations. Numerical data for panels A, B, and C can be found in the file S17 Data. BHC, benzenehexacarboxylic acid; dATP, deoxyadenosine triphosphate; HREX/US, Hamiltonian Replica-exchange/Umbrella Sampling; IP_6_, inositol hexakisphosphate; rNTP, ribonucleoside triphosphate.
(TIF)

S19 Fig Measuring the point stiffness of the HIV-1 capsid by indentation-type experiments. (A) Typical averaged force-distance curve of a core attached to an HMDS pretreated glass slide. For each experiment, approximately 400 such curves were acquired and averaged. Stiffness values were calculated by fitting a linear function to the force curve region bounded by 3- and 4-nm indentation depths. The corresponding line fit is plotted in red. (B) Histogram and Gaussian-fitted curves of the individual measured point stiffness values derived from the consecutive force-distance curves of a single core attached to a glass slide. (C) The individual measured point stiffness values obtained for the core shown in panel A, during a single experiment against the experiment number (count). The point stiffness measurements plot together with an analysis of the narrow distribution of the individual measured spring constants demonstrate that the core did not undergo a significant irreversible deformation during the indentation measurements. Numerical data for panels A, B, and C can be found in the file S18 Data. HMDS, hexamethyldisilazane.
(TIF)

S20 Fig Representative TEM images of mature, immature, eccentric, and dual core virions. Magnification is ×30,000 (scale bar, 100 nm). The total number of WT, K25N, E45A, and K25N/E45A particles counted in each TEM experiment is shown in S7 Table. TEM, transmission electron microscopy; WT, wild-type.
(TIF)

S21 Fig Representative TEM images of mature, immature, eccentric, and dual core virions. Magnification is ×30,000 (scale bar, 100 nm). The total number of WT, K25N, E45A, and K25N/E45A particles counted in each TEM experiment is shown in S7 Table. TEM, transmission electron microscopy; WT, wild-type.
(TIF)

S1 Table Simulations performed in the present study. (XLSX)

S2 Table Distances between dATP and the R18 of each CA monomer in dATP-dATP free energy simulation. (XLSX)

S3 Table Distances between dATP and the R18 of each CA monomer in dATP-IP6 free energy simulation. (XLSX)

S4 Table Distances between dATP and the R18 of each CA monomer in dATP-BHC free energy simulation. (XLSX)

S5 Table Distances between dATP and the K25 of each CA monomer in dATP-dATP free energy simulation. (XLSX)

S6 Table Distances between dATP and the K25 of each CA monomer in dATP-IP6 free energy simulation. (XLSX)

S7 Table Distances between dATP and the K25 of each CA monomer in dATP-BHC free energy simulation. (XLSX)

S8 Table The total number of WT, K25N, E45A, and K25N/E45A particles counted in each TEM experiment in table form. (XLSX)

S1 Movie HIV-1 CA accommodating 2 nucleotides in the central pore. (MP4)

S2 Movie dNTP translocating through an occupied HIV-1 capsid pore with IP6 present. (MP4)

S1 Data Architecture of the HIV-1 CA hexameric cavity. Numerical data for panel D in Fig 1.
(XLSX)

S2 Data Cooperative binding of small molecules to the central hexamer cavity. Numerical data for panels A, C, and E in Fig 2.
(XLSX)

S3 Data Characterization of K25N HIV-1 and K25N/E45A HIV-1. Numerical data for panels A, B, C, D, and E in Fig 3.
(XLSX)

S4 Data Morphologies and mechanical properties of HIV-1 capsids from AFM. Numerical data for panels A and B in Fig 4.
(XLSX)

S5 Data The 1D free energy landscapes of small molecules interacting with CA hexamers (PDBID: 4XFX). Numerical data for panels A, B, C, and D in S1 Fig.
(XLSX)

S6 Data Free energy landscape of cooperative interactions between small molecules and the CA hexamer. Numerical data for panels A, B, C, and D in S3 Fig.
(XLSX)

S7 Data Closest interligand distances observed in the present study. Numerical data for the plot in S4 Fig.
(XLSX)

S8 Data Mean number of water molecules interacting with dNTPs. Numerical data for panels A, B, C, D, and E in S5 Fig.
(XLSX)

S9 Data IP6 and BHC exhibit distinct interactions with CA hexamers. Numerical data for panels E, F, and G in S6 Fig.
(XLSX)

S10 Data CA retention imaging assay distinguishes WT, hyperstable, and hypostable HIV-1 capsid phenotypes. Numerical data for panels A and B in S9 Fig.
(XLSX)

S11 Data The effect of IP6 on HIV-1 CA retention. Numerical data for panels A, B, and C in S10 Fig.
(XLSX)

S12 Data Binding of IP6 to CA pentamers, namely PDBIDs 5MCY and 3P05. Numerical data for panel C in S11 Fig.
(XLSX)

S13 Data Convergence of MD simulations employed in the present study. Numerical data for panels A, B, and C in S14 Fig.
(XLSX)

S14 Data Setup of the 2D HREX/US simulations. Numerical data for panels A, B, and C in S15 Fig.
(XLSX)

S15 Data Convergence of the 1D HREX/US simulations. Numerical data for all panels of S16 Fig.
(XLSX)

S16 Data Convergence of the 2D HREX/US simulations. Numerical data for panels A, B, C, and D in S17 Fig.
(XLSX)

S17 Data Exchange ratios for the HREX/US simulations performed in the present study. Numerical data for panels A, B, and C in S18 Fig.
(XLSX)

S18 Data Measuring the point stiffness of the HIV-1 capsid by indentation-type experiments. Numerical data for panels A, B, and C in S19 Fig.
(XLSX)

## Abbreviations

AFM: atomic force microscopy
BHC: benzenehexacarboxylic acid
dNTP: deoxynucleotide triphosphate
F-D: force-distance
GM-CSF: granulocyte macrophage colony-stimulating factor
HMDS: hexamethyldisilazane
HEK: human embryonic kidney
HREX/US: Hamiltonian Replica-exchange/Umbrella Sampling
IL-2: interleukin-2
IN: integrase
IP: inositol phosphate
IP_6_: inositol hexakisphosphate
LTR: long terminal repeat
MAS: magic angle spinning
MD: molecular dynamics
PBMC: peripheral blood mononuclear cell
PEI: polyethylenimine
PES: Polyethersulfone
PFA: paraformaldehyde
PHA: phytohaemagglutinin
PMF: potential of mean force
PV: progress variable
QI: quantitative imaging
qPCR: quantitative PCR
RMSE: root mean squared error
RT: reverse transcriptase
SMD: steered MD
ssRNA: single-stranded RNA
TEM: transmission electron microscopy
vDNA: viral DNA
WHAM: weighed histogram analysis method
WT: wild-type

## References

[1] Ganser BK, Li S, Klishko VY, Finch JT, Sundquist WI. Assembly and analysis of conical models for the HIV-1 core. Science. 1999;283(5398):80–3. Epub 1999/01/05. 10.1126/science.283.5398.80 . 9872746

[2] Mattei S, Glass B, Hagen WJ, Kräusslich H-G, Briggs JA. The structure and flexibility of conical HIV-1 capsids determined within intact virions. Science. 2016;354 (6318):1434–7. 10.1126/science.aah4972 27980210

[3] Zhao G, Perilla JR, Yufenyuy EL, Meng X, Chen B, Ning J, et al Mature HIV-1 capsid structure by cryo-electron microscopy and all-atom molecular dynamics. Nature. 2013;497(7451):643–6. 10.1038/nature12162 23719463 PMC3729984

[4] Perilla JR, Schulten K. Physical properties of the HIV-1 capsid from all-atom molecular dynamics simulations. Nat Commun. 2017;8:15959 10.1038/ncomms15959 28722007 PMC5524983

[5] Bejarano DA, Peng K, Laketa V, Börner K, Jost KL, Lucic B, et al HIV-1 nuclear import in macrophages is regulated by CPSF6-capsid interactions at the nuclear pore complex. elife. 2019;8:e41800 10.7554/eLife.41800 30672737 PMC6400501

[6] Ambrose Z, Aiken C. HIV-1 uncoating: connection to nuclear entry and regulation by host proteins. Virology. 2014;454:371–9. 10.1016/j.virol.2014.02.004 24559861 PMC3988234

[7] Campbell EM, Hope TJ. HIV-1 capsid: the multifaceted key player in HIV-1 infection. Nat Rev Microbiol. 2015;13(8):471–83. Epub 2015/07/17. 10.1038/nrmicro3503 26179359 PMC4876022

[8] Jacques DA, McEwan WA, Hilditch L, Price AJ, Towers GJ, James LC. HIV-1 uses dynamic capsid pores to import nucleotides and fuel encapsidated DNA synthesis. Nature. 2016;536(7616):349–53. 10.1038/nature19098 27509857 PMC4998957

[9] Perilla JR, Gronenborn AM. Molecular Architecture of the Retroviral Capsid. Trends Biochem Sci. 2016;41(5):410–20. 10.1016/j.tibs.2016.02.009 27039020 PMC4879823

[10] Yamashita M, Engelman AN. Capsid-dependent host factors in HIV-1 infection. Trends Microbiol. 2017;25 (9):741–55. 10.1016/j.tim.2017.04.004 28528781 PMC5562514

[11] Dick RA, Mallery DL, Vogt VM, James LC. IP6 Regulation of HIV Capsid Assembly, Stability, and Uncoating. Viruses. 2018;10(11):640 10.3390/v10110640 30445742 PMC6267275

[12] Mallery DL, Marquez CL, McEwan WA, Dickson CF, Jacques DA, Anandapadamanaban M, et al IP6 is an HIV pocket factor that prevents capsid collapse and promotes DNA synthesis. Elife. 2018;7:e35335 10.7554/eLife.35335 29848441 PMC6039178

[13] Kohlstaedt LA, Wang J, Friedman JM, Rice PA, Steitz TA. Crystal structure at 3.5 A resolution of HIV-1 reverse transcriptase complexed with an inhibitor. Science. 1992;256(5065):1783–90. 10.1126/science.1377403 . 1377403

[14] Campbell S, Fisher RJ, Towler EM, Fox S, Issaq HJ, Wolfe T, et al Modulation of HIV-like particle assembly in vitro by inositol phosphates. Proc Natl Acad Sci. 2001;98 (19):10875–9. 10.1073/pnas.191224698 11526217 PMC58567

[15] Jones CP, Datta SAK, Rein A, Rouzina I, Musier-Forsyth K. Matrix Domain Modulates HIV-1 Gag's Nucleic Acid Chaperone Activity via Inositol Phosphate Binding. J Virol. 2011;85 (4):1594–603. 10.1128/JVI.01809-10 21123373 PMC3028911

[16] Dick RA, Zadrozny KK, Xu C, Schur FKM, Lyddon TD, Ricana CL, et al Inositol phosphates are assembly co-factors for HIV-1. Nature. 2018;560(7719):509–12. 10.1038/s41586-018-0396-4 30069050 PMC6242333

[17] Rankovic S, Varadarajan J, Ramalho R, Aiken C, Rousso I. Reverse Transcription Mechanically Initiates HIV-1 Capsid Disassembly. J Virol. 2017;91(12):e00289–17. 10.1128/JVI.00289-17 28381579 PMC5446659

[18] Hulme AE, Perez O, Hope TJ. Complementary assays reveal a relationship between HIV-1 uncoating and reverse transcription. Proc Natl Acad Sci. 2011;108 (24):9975–80. 10.1073/pnas.1014522108 21628558 PMC3116424

[19] Hadden JA, Perilla JR. All-atom virus simulations. Curr Opin Virol. 2018;31:82–91. 10.1016/j.coviro.2018.08.007 30181049 PMC6456034

[20] Gres AT, Kirby KA, KewalRamani VN, Tanner JJ, Pornillos O, Sarafianos SG. X-ray crystal structures of native HIV-1 capsid protein reveal conformational variability. Science. 2015;349 (6243):99–103. 10.1126/science.aaa5936 26044298 PMC4584149

[21] Rihn SJ, Wilson SJ, Loman NJ, Alim M, Bakker SE, Bhella D, et al Extreme genetic fragility of the HIV-1 capsid. PLoS Pathog. 2013;9(6):e1003461 Epub 2013/07/03. 10.1371/journal.ppat.1003461 23818857 PMC3688543

[22] Lopez CS, Eccles JD, Still A, Sloan RE, Barklis RL, Tsagli SM, et al Determinants of the HIV-1 core assembly pathway. Virology. 2011;417(1):137–46. Epub 2011/06/17. 10.1016/j.virol.2011.05.011 21676426 PMC3152690

[23] Briggs JA, Simon MN, Gross I, Krausslich HG, Fuller SD, Vogt VM, et al The stoichiometry of Gag protein in HIV-1. Nat Struct Mol Biol. 2004;11(7):672–5. Epub 2004/06/23. 10.1038/nsmb785 . 15208690

[24] Francis AC, Marin M, Shi J, Aiken C, Melikyan GB. Time-Resolved Imaging of Single HIV-1 Uncoating In Vitro and in Living Cells. PLoS Pathog. 2016;12 (6):e1005709 10.1371/journal.ppat.1005709 27322072 PMC4913920

[25] Márquez CL, Lau D, Walsh J, Shah V, McGuinness C, Wong A, et al Kinetics of HIV-1 capsid uncoating revealed by single-molecule analysis. elife. 2018;7:e34772 10.7554/eLife.34772 29877795 PMC6039174

[26] Forshey BM, von Schwedler U, Sundquist WI, Aiken C. Formation of a Human Immunodeficiency Virus Type 1 Core of Optimal Stability Is Crucial for Viral Replication. J Virol. 2002;76 (11):5667–77. 10.1128/jvi.76.11.5667-5677.2002 11991995 PMC137032

[27] Ganser-Pornillos BK, von Schwedler UK, Stray KM, Aiken C, Sundquist WI. Assembly Properties of the Human Immunodeficiency Virus Type 1 CA Protein. J Virol. 2004;78 (5):2545–52. 10.1128/jvi.78.5.2545-2552.2004 14963157 PMC369201

[28] Ramalho R, Rankovic S, Zhou J, Aiken C, Rousso I. Analysis of the mechanical properties of wild type and hyperstable mutants of the HIV-1 capsid. Retrovirology. 2016;13(1):17 10.1186/s12977-016-0250-4 26979152 PMC4793510

[29] Zhang H, Dornadula G, Pomerantz RJ. Endogenous reverse transcription of human immunodeficiency virus type 1 in physiological microenviroments: an important stage for viral infection of nondividing cells. J Virol. 1996;70(5):2809–24. 10.1128/JVI.70.5.2809-2824.1996 8627755 PMC190138

[30] Eswar N, Webb B, Marti-Renom MA, Madhusudhan MS, Eramian D, Shen MY, et al Comparative protein structure modeling using Modeller. Curr Protoc Bioinformatics. 2006;Chapter 5(1):Unit-5 6. 10.1002/0471250953.bi0506s15 18428767 PMC4186674

[31] Dolinsky TJ, Czodrowski P, Li H, Nielsen JE, Jensen JH, Klebe G, et al PDB2PQR: expanding and upgrading automated preparation of biomolecular structures for molecular simulations. Nucleic Acids Res. 2007;35(Web Server issue):W522–5. 10.1093/nar/gkm276 17488841 PMC1933214

[32] Dharan A, Bachmann N, Talley S, Zwikelmaier V, Campbell EM. Nuclear pore blockade reveals that HIV-1 completes reverse transcription and uncoating in the nucleus. Nat Microbiol. 2020;5(9):1088–95. Epub 2020/06/03. 10.1038/s41564-020-0735-8 . 32483230 PMC9286700

[33] Berneche S, Roux B. Energetics of ion conduction through the K+ channel. Nature. 2001;414 (6859):73 10.1038/35102067 11689945

[34] Tajkhorshid E, Nollert P, Jensen MØ, Miercke LJ, O'Connell J, Stroud RM, et al Control of the selectivity of the aquaporin water channel family by global orientational tuning. Science. 2002;296 (5567):525–30. 10.1126/science.1067778 11964478

[35] Burdick RC, Li C, Munshi M, Rawson JMO, Nagashima K, Hu WS, et al HIV-1 uncoats in the nucleus near sites of integration. Proc Natl Acad Sci U S A. 2020;117(10):5486–93. Epub 2020/02/26. 10.1073/pnas.1920631117 32094182 PMC7071919

[36] Francis AC, Marin M, Singh PK, Achuthan V, Prellberg MJ, Palermino-Rowland K, et al HIV-1 replication complexes accumulate in nuclear speckles and integrate into speckle-associated genomic domains. Nat Commun. 2020;11(1):3505 Epub 2020/07/16. 10.1038/s41467-020-17256-8 32665593 PMC7360574

[37] Stone JE, Phillips JC, Freddolino PL, Hardy DJ, Trabuco LG, Schulten K. Accelerating molecular modeling applications with graphics processors. J Comput Chem. 2007;28(16):2618–40. 10.1002/jcc.20829 . 17894371

[38] Trabuco LG, Villa E, Schreiner E, Harrison CB, Schulten K. Molecular dynamics flexible fitting: a practical guide to combine cryo-electron microscopy and X-ray crystallography. Methods. 2009;49(2):174–80. 10.1016/j.ymeth.2009.04.005 19398010 PMC2753685

[39] Phillips JC, Braun R, Wang W, Gumbart J, Tajkhorshid E, Villa E, et al Scalable molecular dynamics with NAMD. J Comput Chem. 2005;26(16):1781–802. 10.1002/jcc.20289 16222654 PMC2486339

[40] Stone JE, McGreevy R, Isralewitz B, Schulten K. GPU-accelerated analysis and visualization of large structures solved by molecular dynamics flexible fitting. Faraday Discuss. 2014;169:265–83. 10.1039/c4fd00005f 25340325 PMC4208074

[41] Vanommeslaeghe K, Hatcher E, Acharya C, Kundu S, Zhong S, Shim J, et al CHARMM general force field: A force field for drug-like molecules compatible with the CHARMM all-atom additive biological force fields. J Comput Chem. 2010;31(4):671–90. 10.1002/jcc.21367 19575467 PMC2888302

[42] Vanommeslaeghe K, MacKerell AD Jr. Automation of the CHARMM General Force Field (CGenFF) I: bond perception and atom typing. J Chem Inf Model. 2012;52 (12):3144–54. 10.1021/ci300363c 23146088 PMC3528824

[43] Fiorin G, Klein ML, Henin J. Using collective variables to drive molecular dynamics simulations. Mol Phys. 2013;111(22–23):3345–62. 10.1080/00268976.2013.813594 PubMed PMID: WOS:000327296600006.

[44] Jorgensen WL, Jenson C. Temperature dependence of TIP3P, SPC, and TIP4P water from NPT Monte Carlo simulations: Seeking temperatures of maximum density. J Comput Chem. 1998;19(10):1179–86. 10.1002/(Sici)1096-987x(19980730)19:10&lt;1179::Aid-Jcc6&gt;3.0.Co;2-J PubMed PMID: WOS:000074440800008.

[45] Humphrey W, Dalke A, Schulten K. VMD: visual molecular dynamics. J Mol Graph. 1996;14 (1):33–8. 10.1016/0263-7855(96)00018-5 8744570

[46] Perilla JR, Hadden JA, Goh BC, Mayne CG, Schulten K. All-atom molecular dynamics of virus capsids as drug targets. J Phys Chem Lett. 2016;7 (10):1836–44. 10.1021/acs.jpclett.6b00517 27128262 PMC4876486

[47] Jiang W, Luo Y, Maragliano L, Roux B. Calculation of Free Energy Landscape in Multi-Dimensions with Hamiltonian-Exchange Umbrella Sampling on Petascale Supercomputer. J Chem Theory Comput. 2012;8(11):4672–80. 10.1021/ct300468g . 26605623

[48] Jiang W, Phillips JC, Huang L, Fajer M, Meng Y, Gumbart JC, et al Generalized Scalable Multiple Copy Algorithms for Molecular Dynamics Simulations in NAMD. Comput Phys Commun. 2014;185(3):908–16. 10.1016/j.cpc.2013.12.014 24944348 PMC4059768

[49] Sugita Y, Kitao A, Okamoto Y. Multidimensional replica-exchange method for free-energy calculations. J Chem Phys. 2000;113(15):6042–51. doi: Pii [S0021-9606(00)50739-9] 10.1063/1.1308516. PubMed PMID: WOS:000089635200005.

[50] Fukunishi H, Watanabe O, Takada S. On the Hamiltonian replica exchange method for efficient sampling of biomolecular systems: Application to protein structure prediction. J Chem Phys. 2002;116 (20):9058–67.

[51] Wojtas-Niziurski W, Meng Y, Roux B, Berneche S. Self-Learning Adaptive Umbrella Sampling Method for the Determination of Free Energy Landscapes in Multiple Dimensions. J Chem Theory Comput. 2013;9(4):1885–95. 10.1021/ct300978b 23814508 PMC3694627

[52] Isralewitz B, Izrailev S, Schulten K. Binding pathway of retinal to bacterio-opsin: a prediction by molecular dynamics simulations. Biophys J. 1997;73 (6):2972 10.1016/S0006-3495(97)78326-7 9414212 PMC1181203

[53] Kumar S, Rosenberg JM, Bouzida D, Swendsen RH, Kollman PA. THE weighted histogram analysis method for free-energy calculations on biomolecules. I The method J Comput Chem. 1992;13 (8):1011–21. 10.1002/jcc.540130812

[54] Kumar S, Rosenberg JM, Bouzida D, Swendsen RH, Kollman PA. Multidimensional free-energy calculations using the weighted histogram analysis method. J Comput Chem. 1995;16 (11):1339–50.

[55] Dehart JL, Andersen JL, Zimmerman ES, Ardon O, An DS, Blackett J, et al The ataxia telangiectasia-mutated and Rad3-related protein is dispensable for retroviral integration. J Virol. 2005;79(3):1389–96. 10.1128/JVI.79.3.1389-1396.2005 15650165 PMC544104

[56] Welker R, Hohenberg H, Tessmer U, Huckhagel C, Krausslich HG. Biochemical and structural analysis of isolated mature cores of human immunodeficiency virus type 1. J Virol. 2000;74(3):1168–77. 10.1128/jvi.74.3.1168-1177.2000 10627527 PMC111451

[57] Rankovic S, Ramalho R, Aiken C, Rousso I. PF74 Reinforces the HIV-1 Capsid To Impair Reverse Transcription-Induced Uncoating. J Virol. 2018;92(20):e00845–18. 10.1128/JVI.00845-18 30089694 PMC6158434

[58] Hutter JL, Bechhoefer J. Calibration of atomic-force microscope tips. Rev Sci Instrum. 1993;64 (7):1868–73. 10.1063/1.1143970

[59] Cecilia D, KewalRamani VN, O'Leary J, Volsky B, Nyambi P, Burda S, et al Neutralization profiles of primary human immunodeficiency virus type 1 isolates in the context of coreceptor usage. J Virol. 1998;72(9):6988–96. 10.1128/JVI.72.9.6988-6996.1998 9696790 PMC109918

[60] Bartz SR, Vodicka MA. Production of high-titer human immunodeficiency virus type 1 pseudotyped with vesicular stomatitis virus glycoprotein. Methods. 1997;12 (4):337–42. 10.1006/meth.1997.0487 9245614

[61] Ning J, Zhong Z, Fischer DK, Harris G, Watkins SC, Ambrose Z, et al Truncated CPSF6 forms higher-order complexes that bind and disrupt HIV-1 capsid. J Virol. 2018;92 (13):e00368–18. 10.1128/JVI.00368-18 29643241 PMC6002704

[62] Byeon IJ, Meng X, Jung J, Zhao G, Yang R, Ahn J, et al Structural convergence between Cryo-EM and NMR reveals intersubunit interactions critical for HIV-1 capsid function. Cell. 2009;139(4):780–90. 10.1016/j.cell.2009.10.010 19914170 PMC2782912

[63] Fung B, Khitrin A, Ermolaev K. An improved broadband decoupling sequence for liquid crystals and solids. J Magn Reson. 2000;142 (1):97–101. 10.1006/jmre.1999.1896 10617439

[64] Marion D, Ikura M, Tschudin R, Bax A. Rapid recording of 2D NMR spectra without phase cycling. Application to the study of hydrogen exchange in proteins. J Magn Reson. 1989;85 (2):393–9.

[65] Bennett AE, Rienstra CM, Auger M, Lakshmi K, Griffin RG. Heteronuclear decoupling in rotating solids. J Chem Phys. 1995;103 (16):6951–8.

[66] Lee W, Tonelli M, Markley JL. NMRFAM-SPARKY: enhanced software for biomolecular NMR spectroscopy. Bioinformatics. 2015;31 (8):1325–7. 10.1093/bioinformatics/btu830 25505092 PMC4393527

[67] Perilla JR, Zhao G, Lu M, Ning J, Hou G, Byeon I-JL, et al CryoEM structure refinement by integrating NMR chemical shifts with molecular dynamics simulations. J Phys Chem B. 2017;121 (15):3853–63. 10.1021/acs.jpcb.6b13105 28181439 PMC5459578

[68] Lu M, Russell RW, Bryer AJ, Quinn CM, Hou G, Zhang H, et al Atomic-resolution structure of HIV-1 capsid tubes by magic-angle spinning NMR. Nat Struct Mol Biol. 2020;27 (9):863–9. 10.1038/s41594-020-0489-2 32901160 PMC7490828

