# Supplementary figures and images for: Permeability of the HIV-1 capsid to metabolites modulates viral DNA synthesis
Source: PLoS Biol. 2020 Dec 17;18(12):e3001015. doi: 10.1371/journal.pbio.3001015 (PMC7775124; doi:10.1371/journal.pbio.3001015)

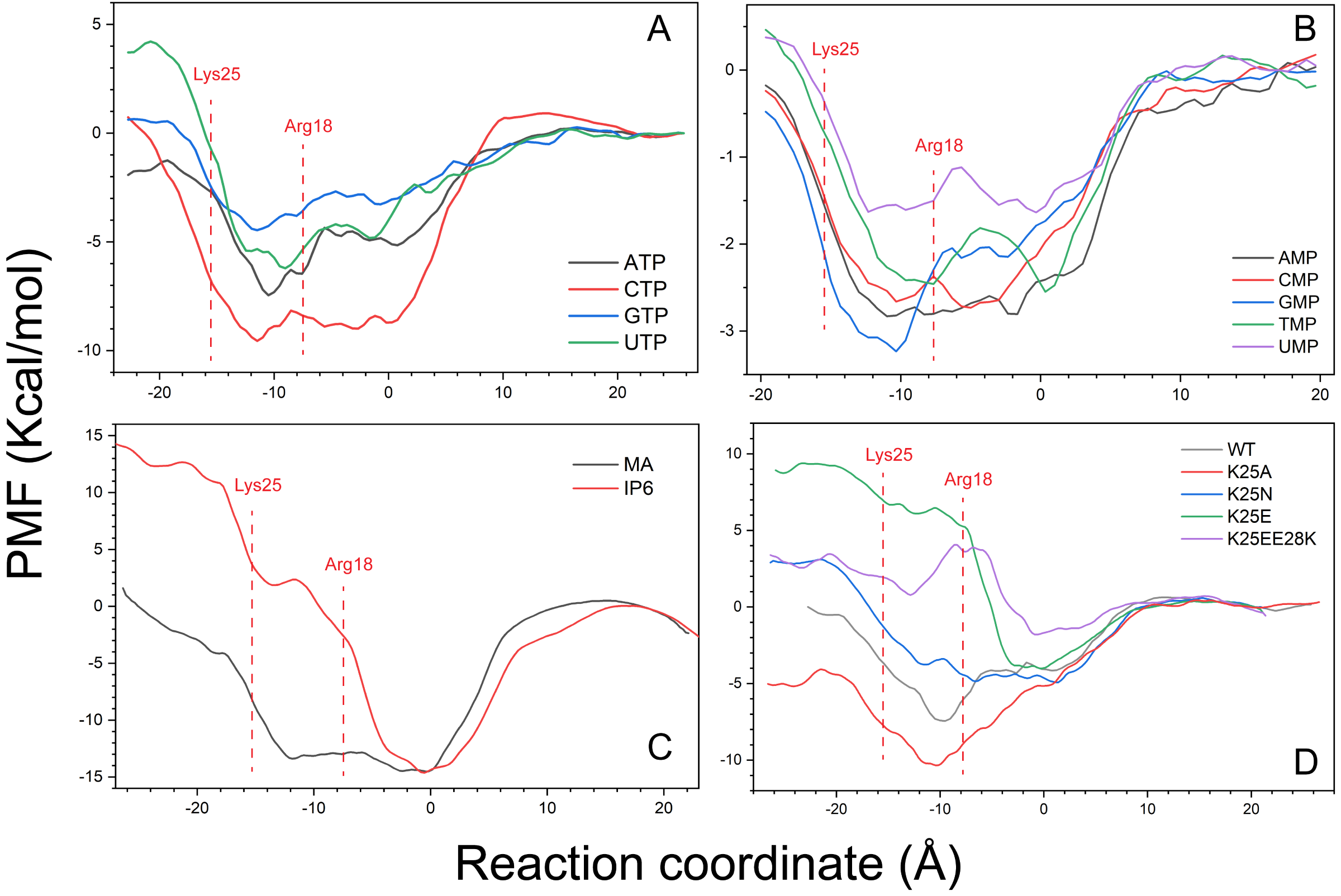

Supplement: S1 Fig — (A) Binding profiles of rNTPs show different binding affinities and specifics compared to dNTPs. (B) Binding profiles of nucleotide monophosphates, which are weaker compared to rNTPs and dNTPs. (C) Binding profile of BHC and IP6 to a hexamer. BHC presents a much wider well compared to IP6. (D) 1D free energy landscapes of dATP binding to WT CA and indicated mutants. Compared to WT, K25A should result in leaky capsids, in contrast to K25N, K25E, and K25E/E28K, which each should result in blocked capsid cavities. Numerical data for panels A, B, C, and D can be found in S5 Data. BHC, benzenehexacarboxylic acid; dATP, deoxyadenosine triphosphate; dNTP, deoxynucleotide triphosphate; IP6, inositol hexakisphosphate; rNTP, ribonucleoside triphosphate; WT, wild-type. (TIF) [file pbio.3001015.s001.tif]

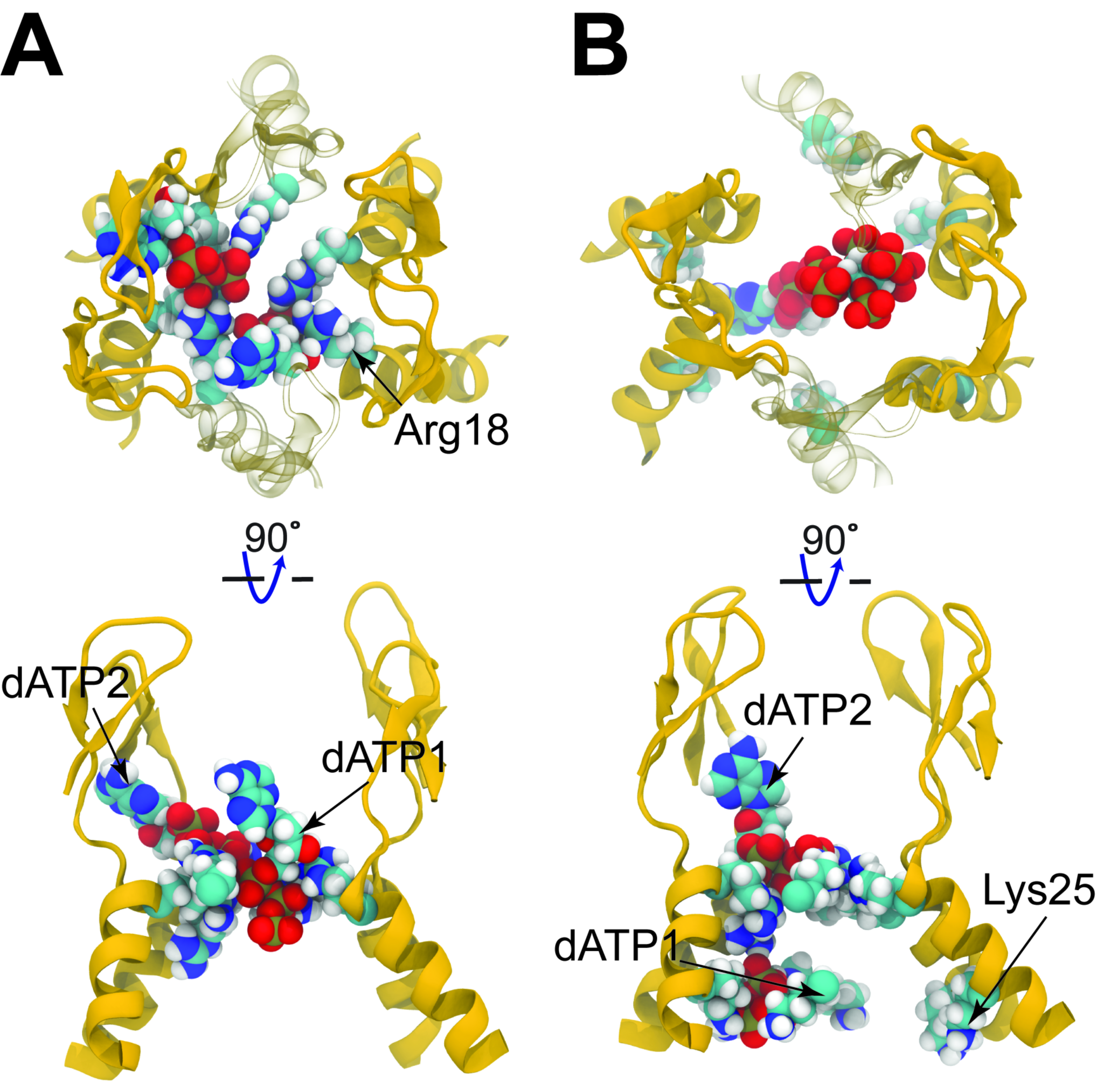

Supplement: S2 Fig — The radius of the spheres represent the van der Waals radii for each nuclei, hydrogens are shown in white, phosphates are shown in yellow, oxygen in red, nitrogen in dark blue, and carbon in cyan. The NTD domain of CA is shown in yellow, illustrating helix 1 and the beta-hairpin region. (A) Two dATP molecules near R18 are shown. (B) Two dATP molecules one above R18 and the second below, interacting with K25 are shown. dATP, deoxyadenosine triphosphate; NTD, N terminal domain. (TIF) [file pbio.3001015.s002.tif]

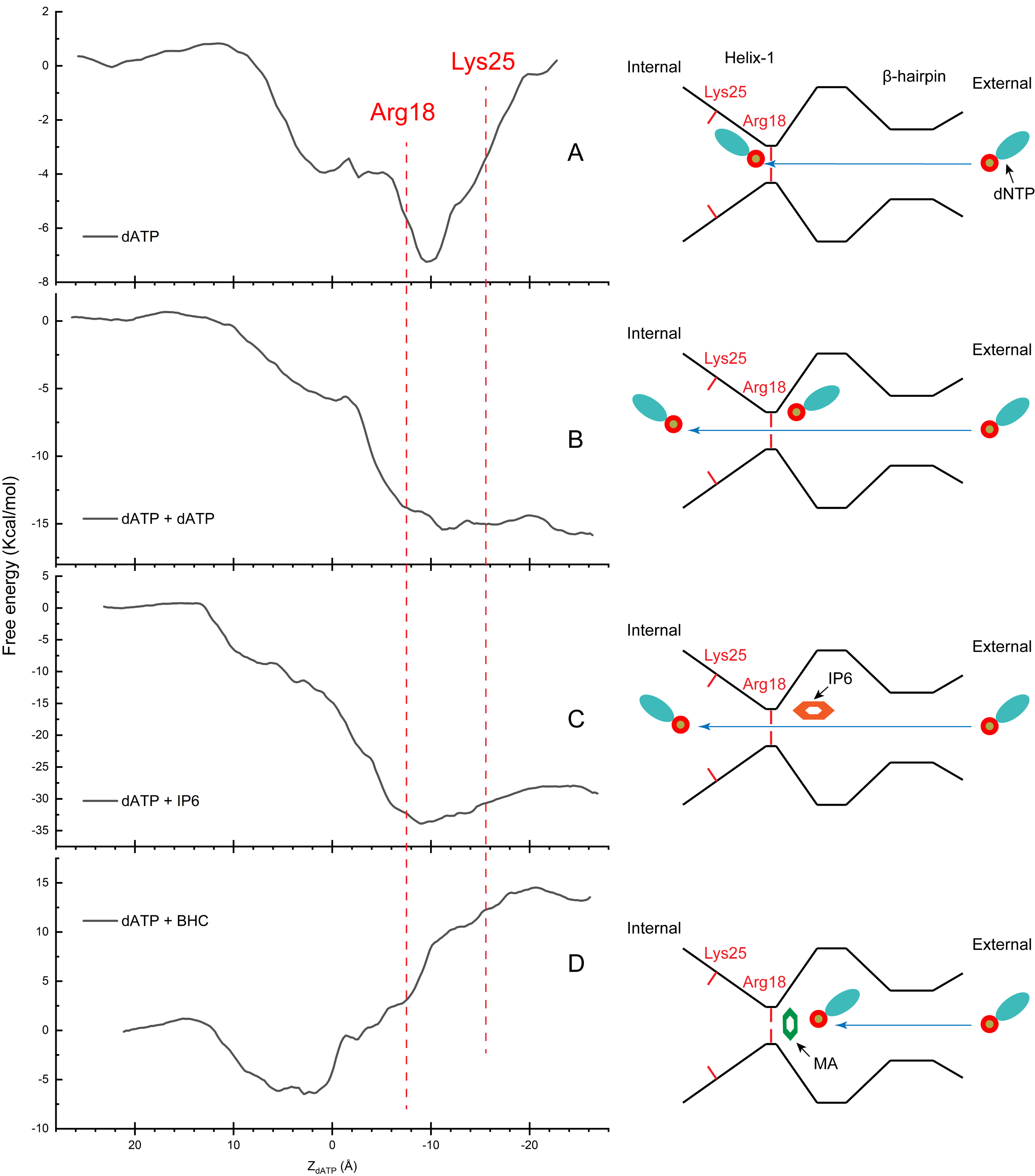

Supplement: S3 Fig — (A) Free energy profile for a single dATP interacting with CA. The profile indicates dNTP binding, but not translocation as the energy barrier is too high to be overcome by simple thermal fluctuations. (B) Cooperation between multiple dATP molecules shifts the free energy profile from binding to a gradient pointing toward the interior of the capsid. (C) Cooperation between IP6 and dNTPs also creates a gradient toward the interior, but almost twice as strong compared to dNTPs alone. (D) BHC inside of the CA cavity facilitates dNTP binding but not translocation. Numerical data for panels A, B, C and D can be found in S6 Data. BHC, benzenehexacarboxylic acid; dATP, deoxyadenosine triphosphate; dNTP, deoxynucleotide triphosphate; IP6, inositol hexakisphosphate. (TIF) [file pbio.3001015.s003.tif]

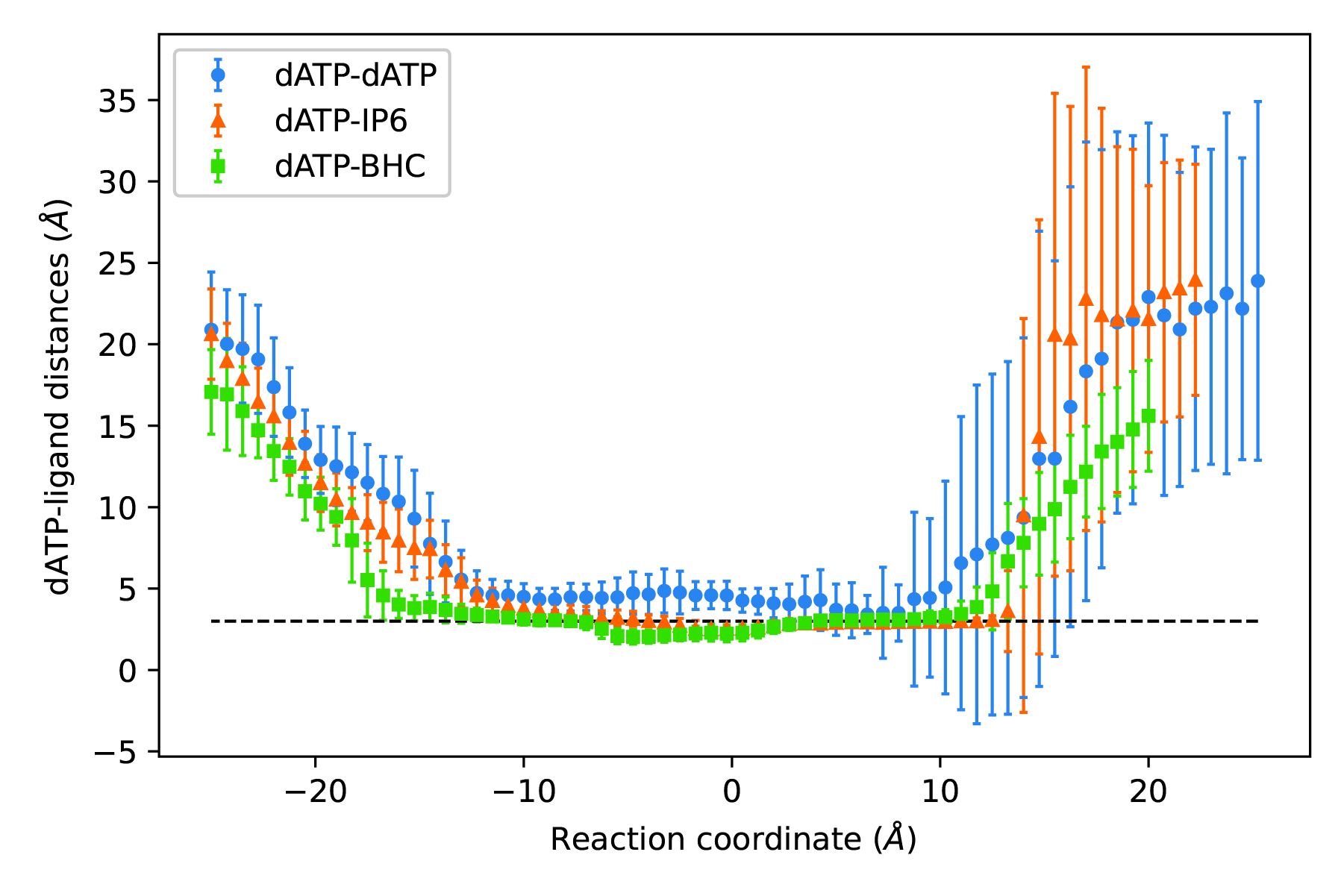

Supplement: S4 Fig — Numerical data for the plot can be found in S7 Data. (TIF) [file pbio.3001015.s004.tif]

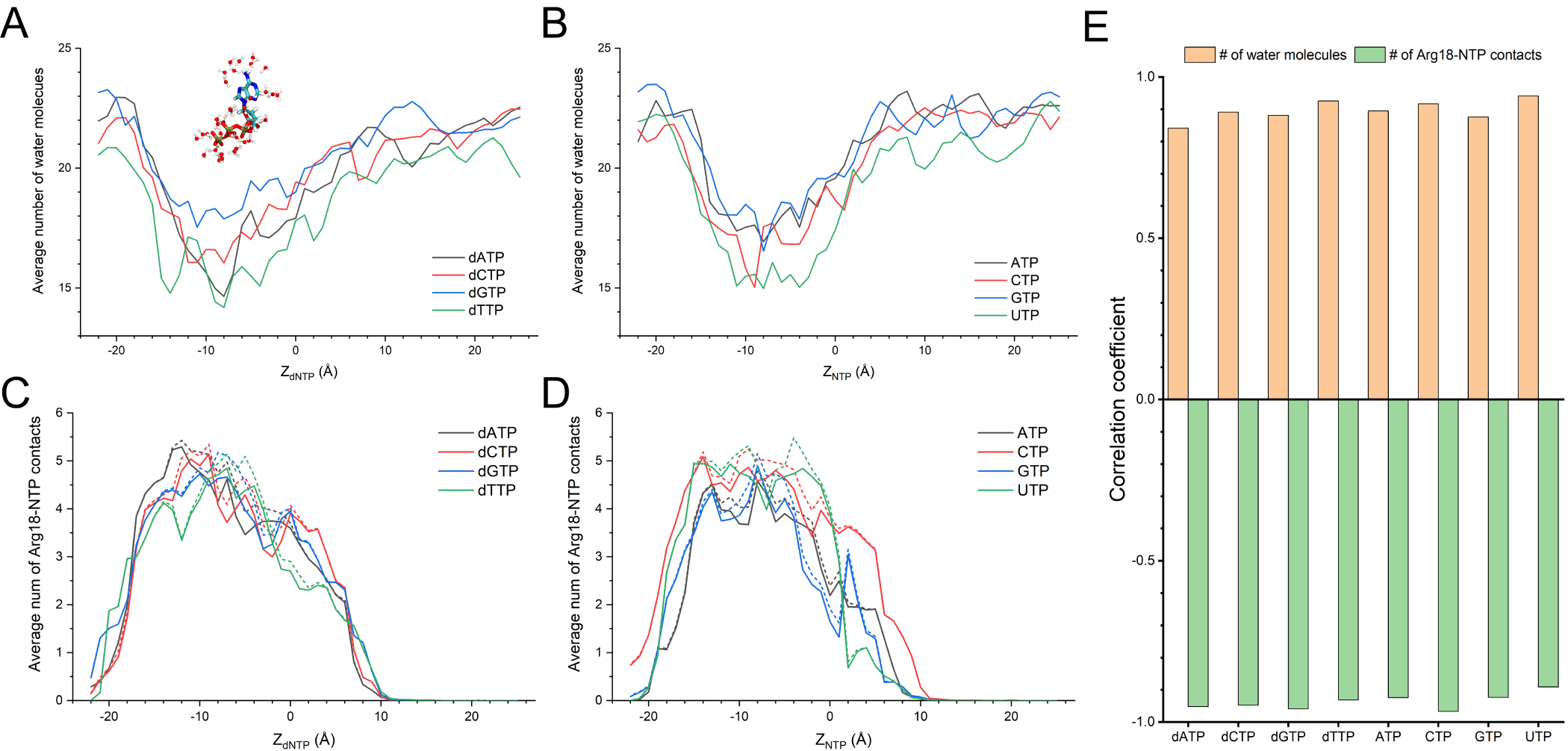

Supplement: S5 Fig — (A) A significant dewetting of dNTPs is observed near Arg18, indicating a loss of conformational entropy. (B) Similarly, loss of water molecules is observed for rNTPs. The loss of entropy is compensated by the formation of salt-bridges between Arg18 and dNTPs (C) and rNTPs (D). (E) Correlation analysis between loss of solvation molecules and formation of bonds with Arg18 and NTPs. Importantly, solvation of the dNTP/rNTP as it moves toward the interior of the capsid is assisted by several charged or polar residues including K25/30 and E28/29. Numerical data for panels A, B, C, D, and E can be found in S8 Data. dNTP, deoxynucleotide triphosphate; NTP, nucleoside triphosphate; rNTP, ribonucleoside triphosphate. (TIF) [file pbio.3001015.s005.tif]

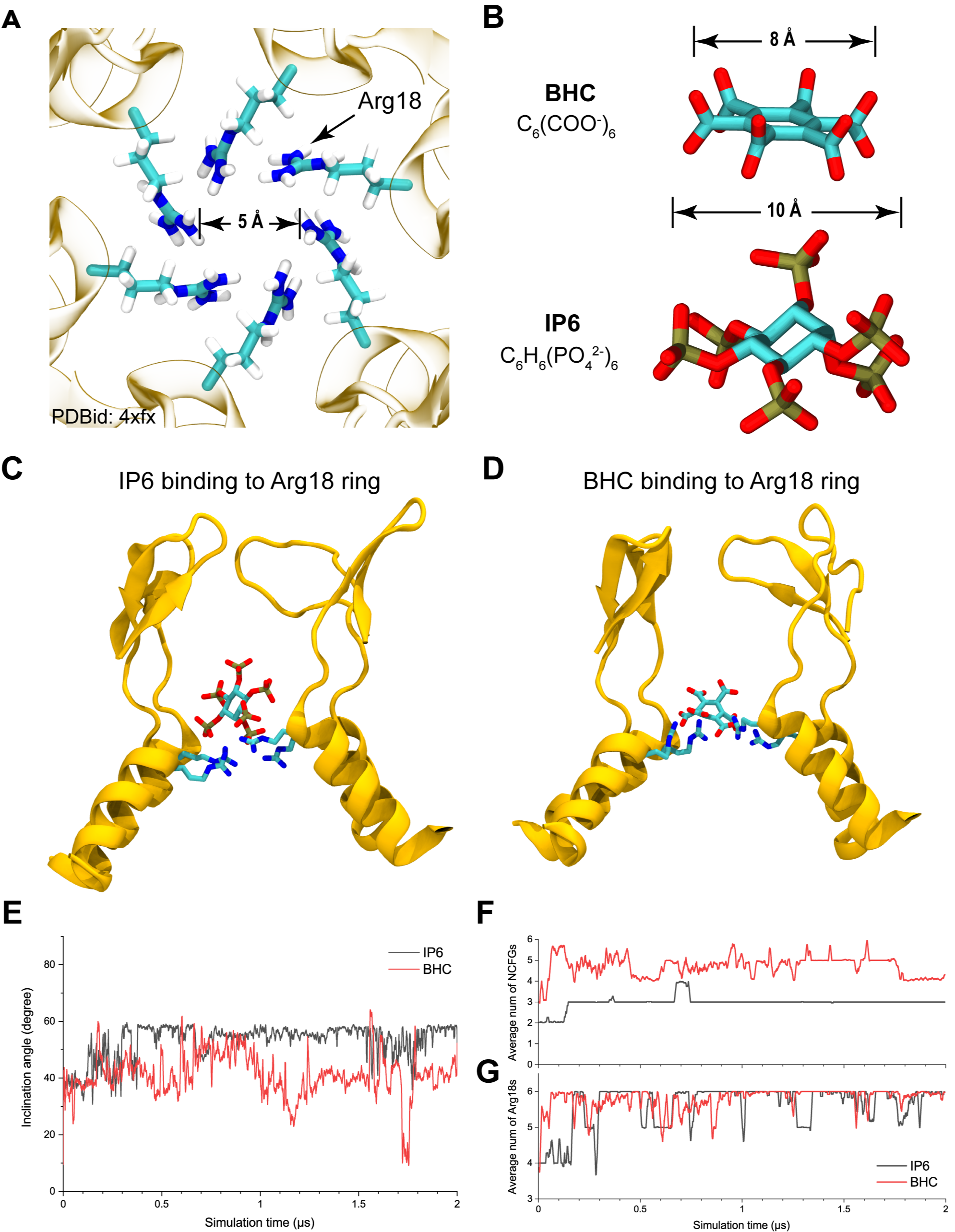

Supplement: S6 Fig — (A) Molecular architecture of the R18 ring in hexamers as observed in the crystal structure (PDBID: 4xfx). (B) Molecular structure of BHC and myo-IP6. (C) Binding of IP6 and (D) BHC to the ring of R18. (E) The angle of inclination is defined as the angle between the plane defined by the 6-membered rings and the plane of the CA-hexamer. Time evolution of the inclination angle for IP6 and BHC. The 2 molecules exhibit different inclination angles. (F) NCFGs within 3.4 Å of R18. (G) Number of R18 within 3.4 Å of IP6 and BHC. Numerical data for panels E, F, and G can be found in S9 Data. BHC, benzenehexacarboxylic acid; IP6, inositol hexakisphosphate; NCFG, number of negatively charged functional group. (TIF) [file pbio.3001015.s006.tif]

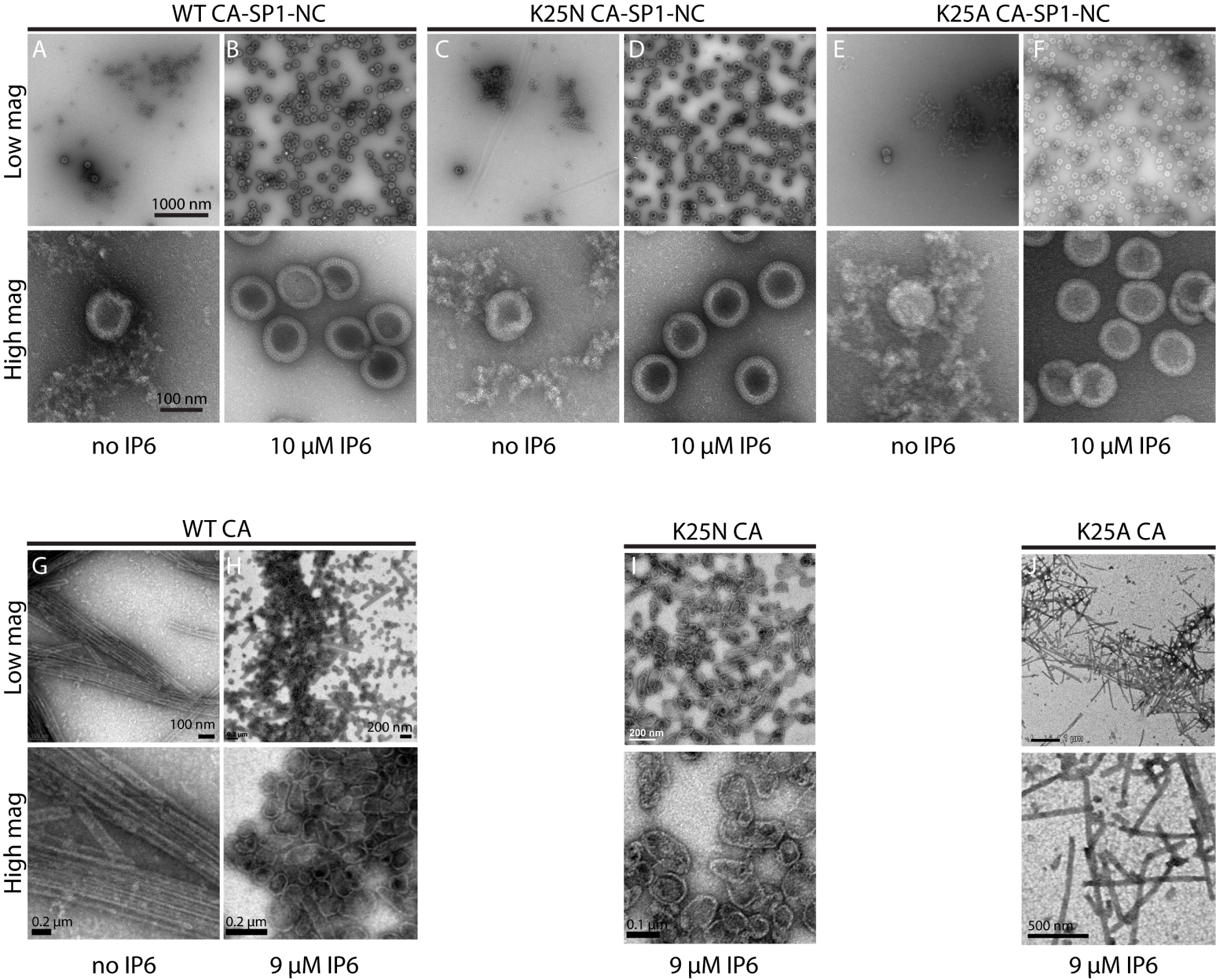

Supplement: S7 Fig — (A–F) Negatively stained TEM images of WT (A, B), K25N (C, D) and K25A (E, F) CA-SP1-NC. The mutants produce virus-like particles with immature morphologies. Assemblies in (A, C, E) contain 2 mg/ml of the respective protein in 50 mM Tris-HCl (pH 8.0), 100 mM NaCl. Assemblies in (B, D, F) contain 10 mg/ml of the respective protein in 50 mM MES (pH 6.0), 10 μM IP6. (G–J) Negatively stained TEM images of WT (G, H) K25N (I) and K25N (J) CA show virus-like particles of mature morphology, namely tubes and cones. Assemblies in panel (G) contain 20 mg/ml CA in 50 mM Tris-HCl (pH 8.0), 2.4 M NaCl. Assemblies in panels H, L, and J contain 20 mg/ml of the respective protein in 50 mM MES (pH 6.0), 9 μM IP6. TEM, transmission electron microscopy; WT, wild-type. (TIF) [file pbio.3001015.s007.tif]

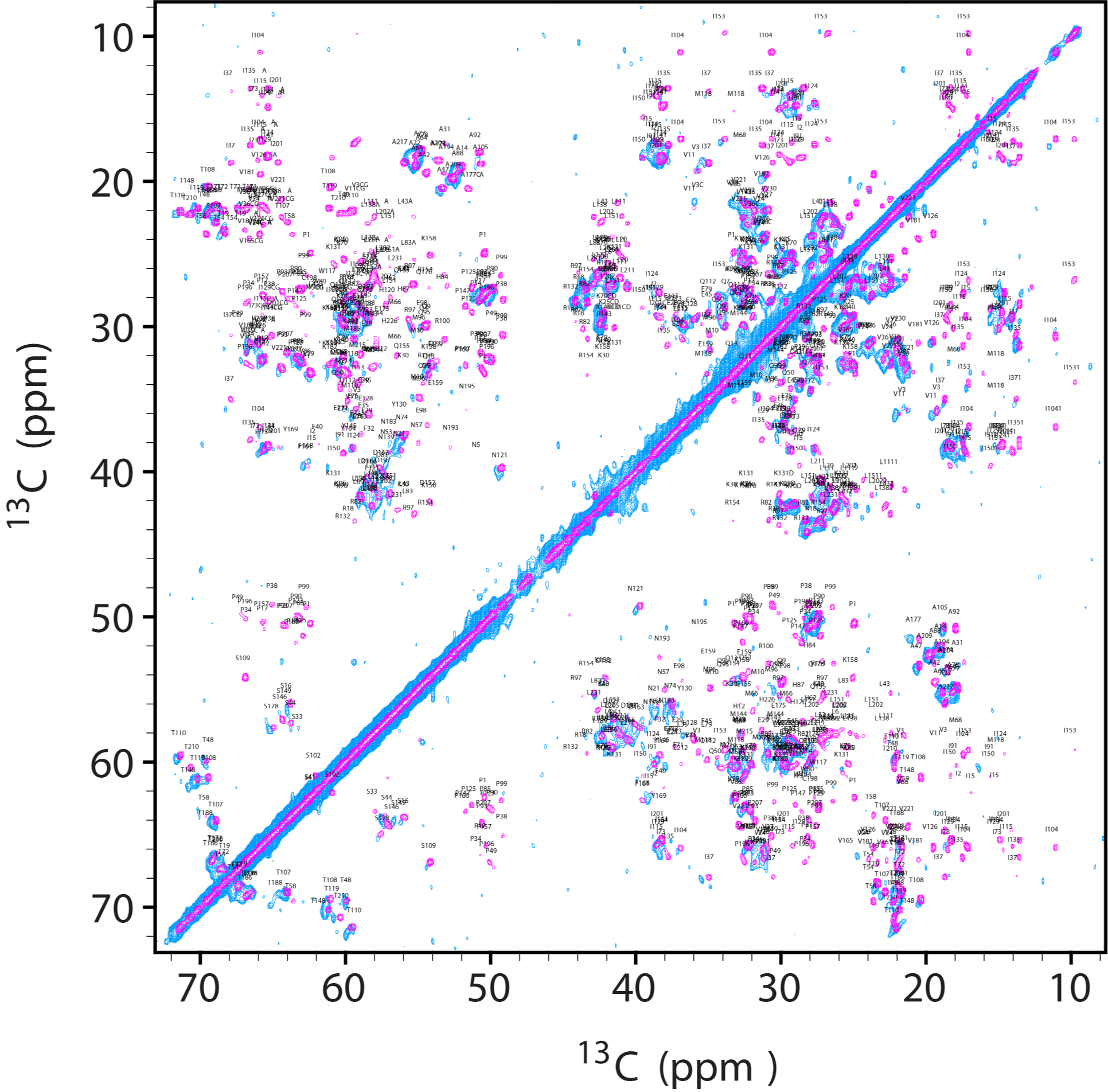

Supplement: S8 Fig — The assemblies contain 20 mg/ml of the respective protein in 50 mM MES (pH 6.0), 0.9 μM IP6. The similar chemical shifts indicate that K25N mutant is folded and its overall structure is the same as in the WT CA conical assemblies. CORD, combined R2vn-driven spin diffusion; IP6, inositol hexakisphosphate; MAS, magic angle spinning; RFDR, homonuclear radio-frequency driven recoupling; WT, wild-type. (TIF) [file pbio.3001015.s008.tif]

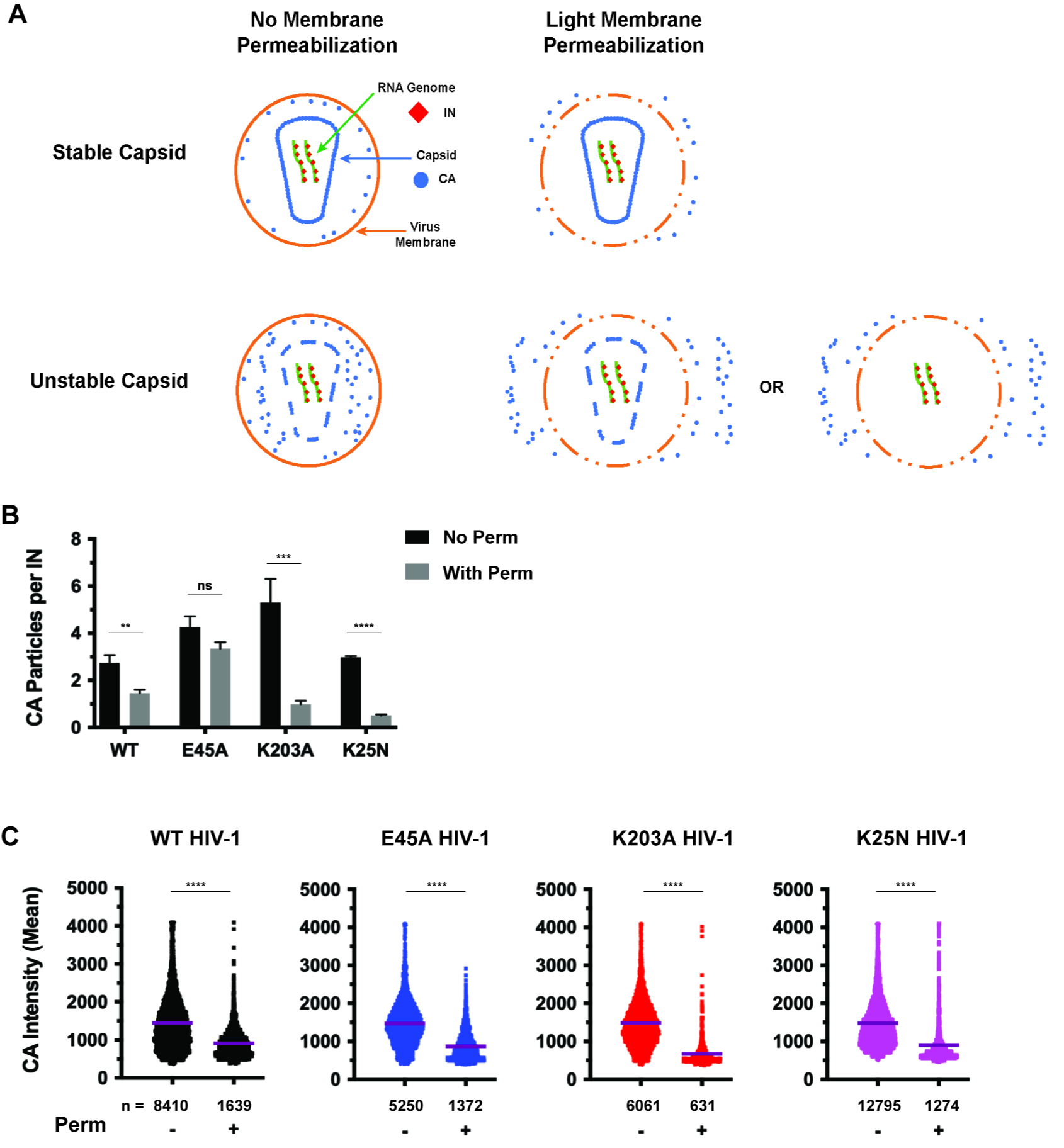

Supplement: S9 Fig — (A) Schematic of the in vitro capsid stability assay. An intact virus membrane (without permeabilization, left) traps loose CA, such that a stable and unstable capsid have similar stained CA fluorescence signals. Upon light permeabilization of the virus membrane (right), loose CA can diffuse from the virus particle and does not contribute to the stained CA fluorescence signal. Fluorescently labeled IN associated with the RNA genome remains trapped within the lightly permeabilized virus membrane. Comparing the IN-normalized CA staining retention upon light permeabilization permits differentiation of stable and unstable capsid. (B) Viruses packaging mRuby3-IN in trans with WT CA or CA bearing the indicated mutations were captured on glass either with or without prefixation virus membrane permeabilization, immunostained for CA, and imaged. The number of CA particles per IN particles is shown for each virus +/− permeabilization. E45A and K203A HIV-1 are included as examples of hyperstable and hypostable CA mutants, respectively. Error bars indicate SEM for 2 experiments. (C) The mean fluorescence intensity of CA staining for each imaged virus particle is shown, with the overall mean intensity for the population indicated by the purple bar. Representative results are shown from 1 experiment. ** P < 0.01; *** P < 0.001; **** P < 0.0001. Numerical data for panels A and B can be found S10 Data. IN, integrase; WT, wild-type. (TIF) [file pbio.3001015.s009.tif]

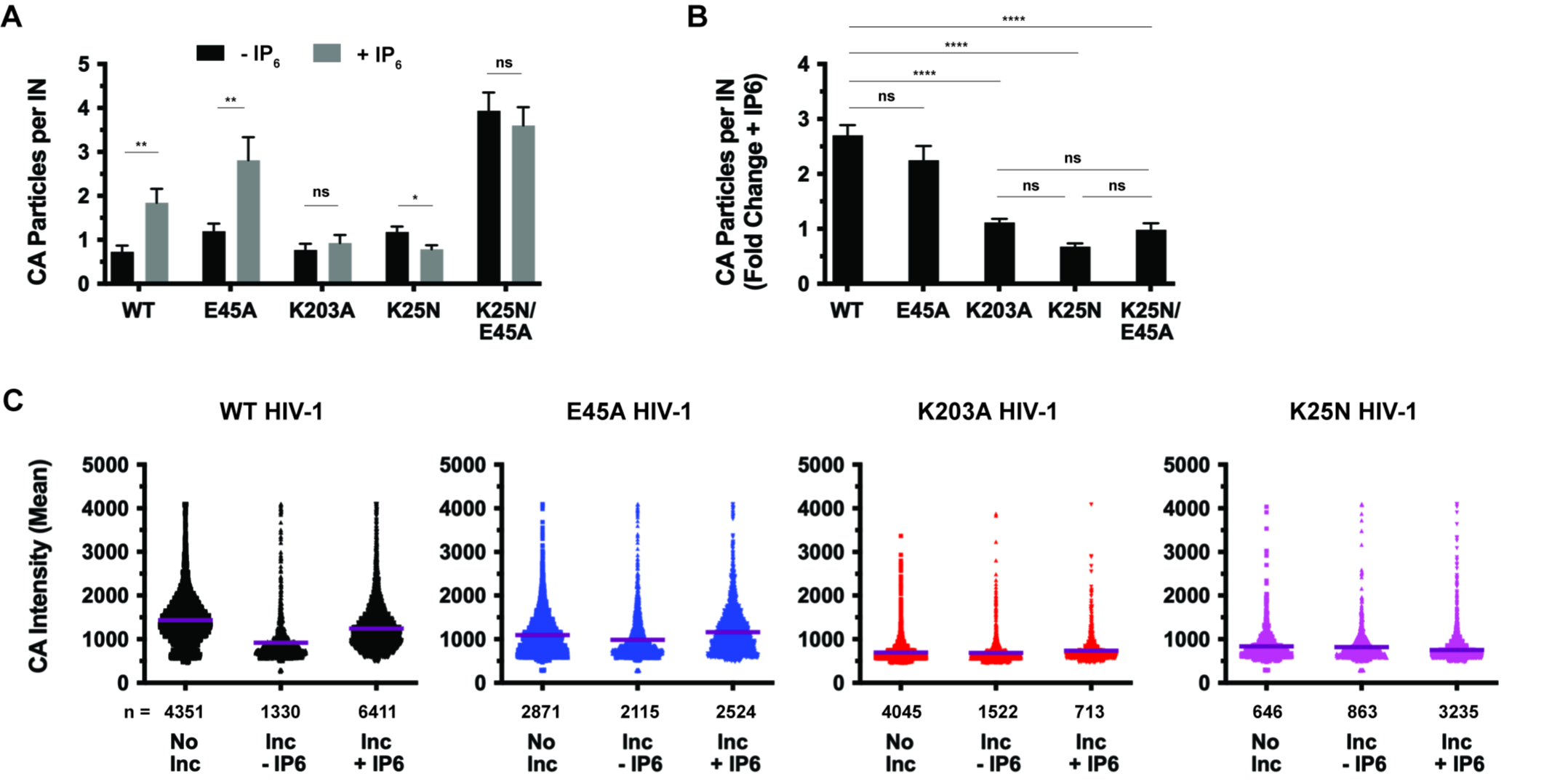

Supplement: S10 Fig — WT or mutant viruses containing mRuby3-IN were captured on glass, lightly permeabilized, and incubated for 2 h in STE buffer with or without 100 μM IP6. Viruses were lightly permeabilized again, fixed, immunostained for CA, and imaged. (A) The number of CA particles per IN particles is shown for each virus +/− IP6. Error bars indicate SEM for 2 experiments. (B) The data from panel A are shown expressed as the fold change of CA staining retained for each virus with the addition of IP6 during incubation. (C) The mean fluorescence intensity of CA staining for each imaged virus particle is shown, with the overall mean intensity for the population indicated by the purple bar. Representative results are shown from 1 experiment. * P < 0.05; ** P < 0.01; *** P < 0.001; **** P < 0.0001. Numerical data for panels A, B, and C can be found in S11 Data. IN, integrase; IP6, inositol hexakisphosphate; WT, wild-type. (TIF) [file pbio.3001015.s010.tif]

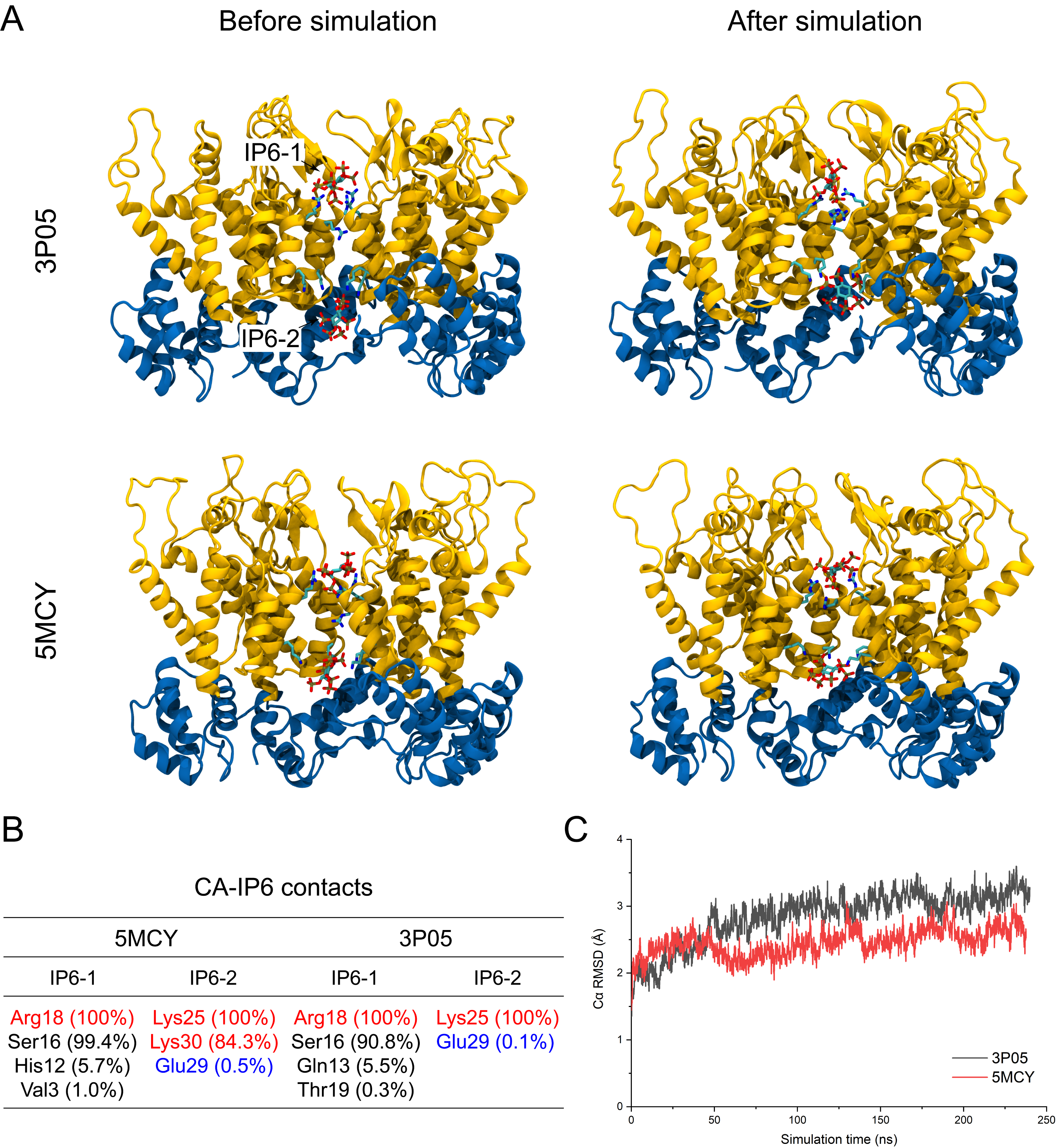

Supplement: S11 Fig — (A) Models based on the crystallographic cross-linked pentamer, and the cryoEM-derived model from intact HIV-1 particles. The figures illustrate the structures of IP6-bound CA pentamers before and after MD simulations. (B) Contact occupancy between CA pentamer residues and IP6. (C) Traces of the root mean squared deviations for alpha carbons in the CA pentamer models. The low RMSD illustrate that IP6 stabilizes both structures. Numerical data for panel C can be found in S12 Data. cryoEM, cryogenic electron microscopy; IP6, inositol hexakisphosphate; MD, molecular dynamics; RMSD, root-mean-square deviation. (TIF) [file pbio.3001015.s011.tif]

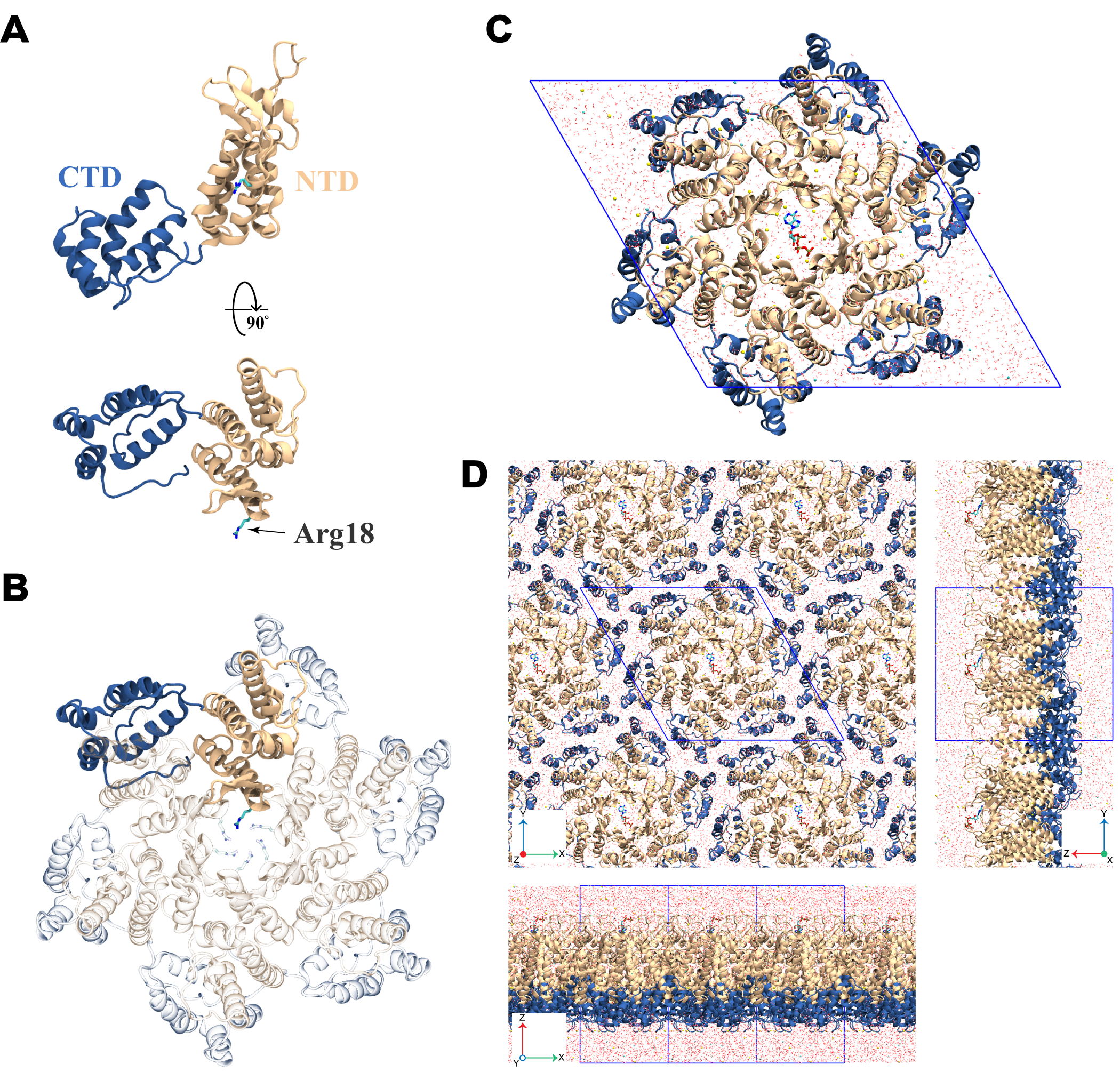

Supplement: S12 Fig — (A) The structure of an HIV-1 CA monomer and (B) hexamer. (C) The CA hexamer in a hexagonal water box with 150 mM NaCl and dATP. (D) A flat CA hexamer lattice formed by applying periodic boundary conditions. (TIF) [file pbio.3001015.s012.tif]

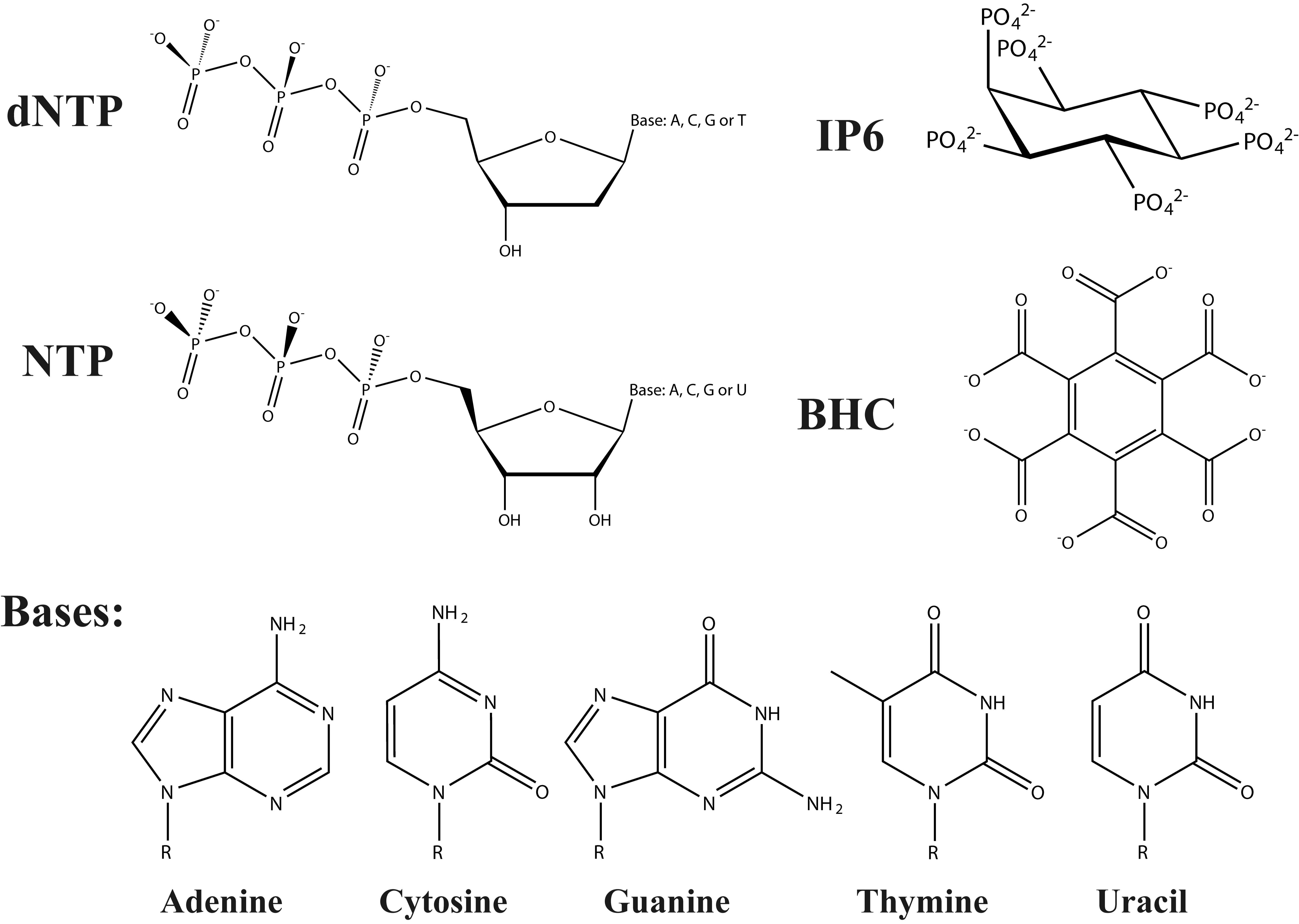

Supplement: S13 Fig — dNTP, rNTP, myo-IP6, BHC, and dNTP and rNTP bases. BHC, benzenehexacarboxylic acid; dNTP, deoxynucleotide triphosphate; rNTP, ribonucleoside triphosphate. (TIF) [file pbio.3001015.s013.tif]

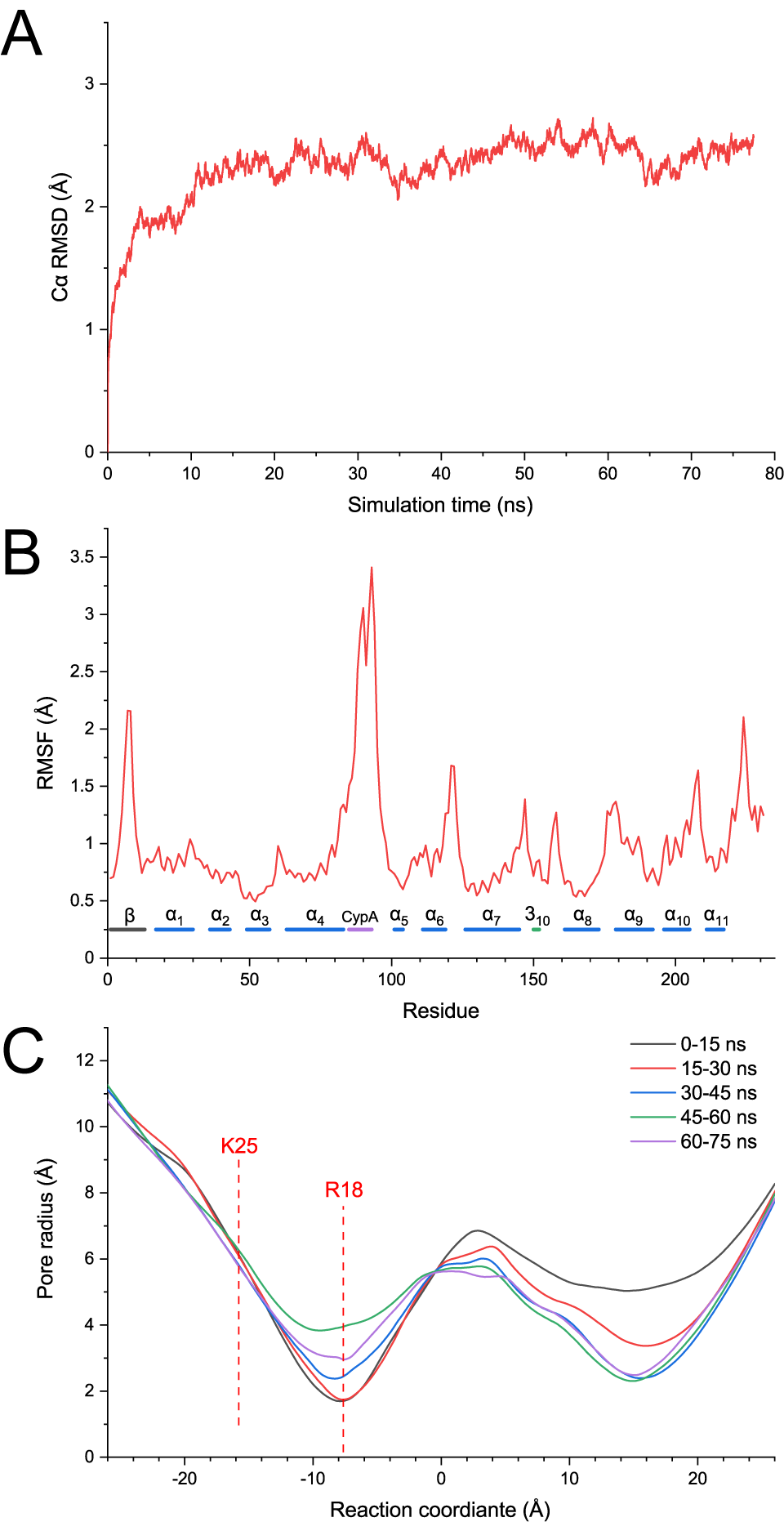

Supplement: S14 Fig — (A) RMSD trace as a function of time during the equilibration phase of the systems. (B) RMSF of CA monomers during equilibration of the systems. (C) Convergence of the radius of the CA hexamers pores. Numerical data for panels A, B, and C can be found in the file S13 Data. MD, molecular dynamics; RMSD, root-mean-square deviation; RMSF, root-mean-square fluctuations. (TIF) [file pbio.3001015.s014.tif]

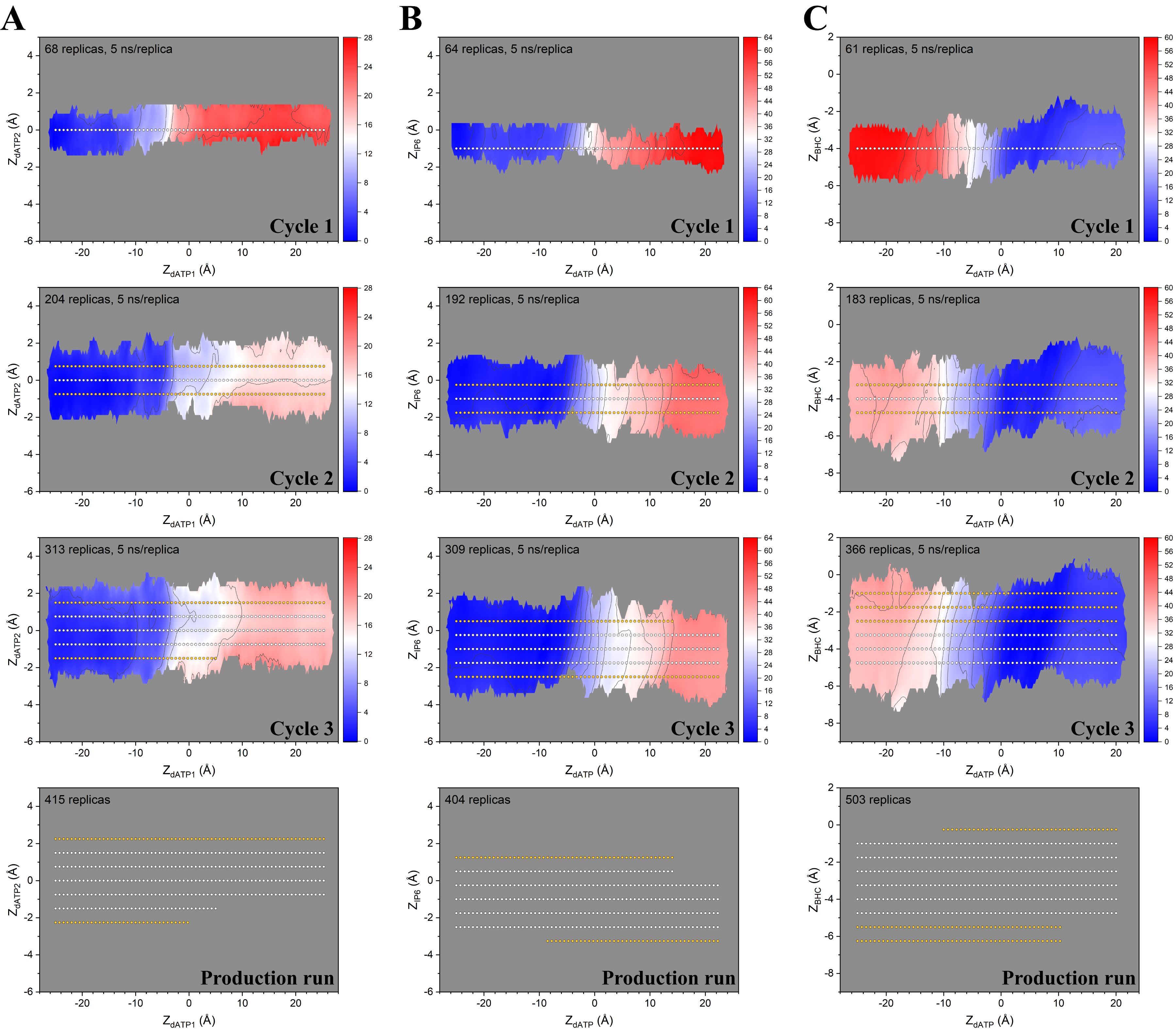

Supplement: S15 Fig — (A) Generation of initial seeds for 2D HREX/US simulations of the 2 dATP models, (B) a dATP with IP6 and (C) BHC model, through 3 cycles of 5 ns HREX/US simulations. In each cycle, new US windows were generated to increase the sampling in 2D space. The initial conformations of the first cycles were extracted from SMD simulations pulling dATP through the hexamer central pore. After that, the initial conformations for new US windows (shown as yellow dots) were copied from the last frame of nearest previous US windows (shown as white dots). After 3 cycles, the generated seeds were used in 30 ns production runs. Numerical data for panel A, B and C can be found in the file S14 Data. BHC, benzenehexacarboxylic acid; dATP, deoxyadenosine triphosphate; HREX/US, Hamiltonian Replica-exchange/Umbrella Sampling; IP6, inositol hexakisphosphate; SMD, steered MD. (TIF) [file pbio.3001015.s015.tif]

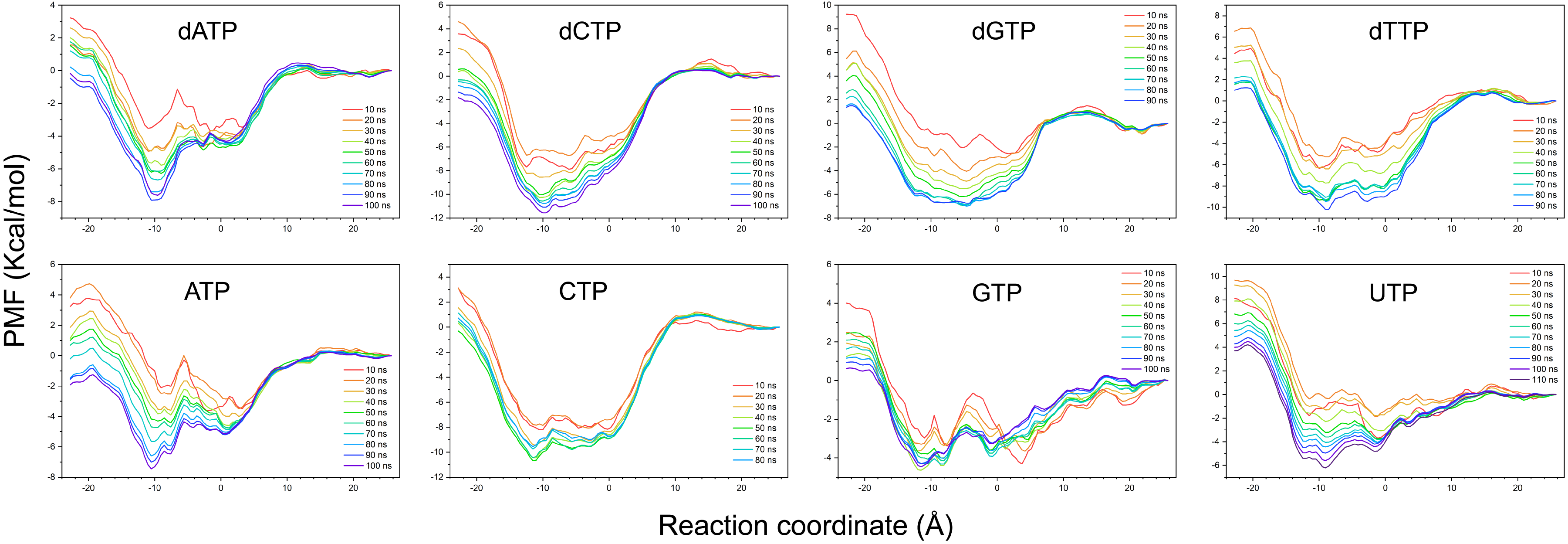

Supplement: S16 Fig — Sequential changes of PMF from every 10 ns HREX/US simulations are shown. After 10 ns, the PMF profiles were used to compute the 1D PMF for NTP translocation. Numerical data for all panels can be found in the file S15 Data. HREX/US, Hamiltonian Replica-exchange/Umbrella Sampling; NTP, nucleoside triphosphate; PMF, potential of mean force. (TIF) [file pbio.3001015.s016.tif]

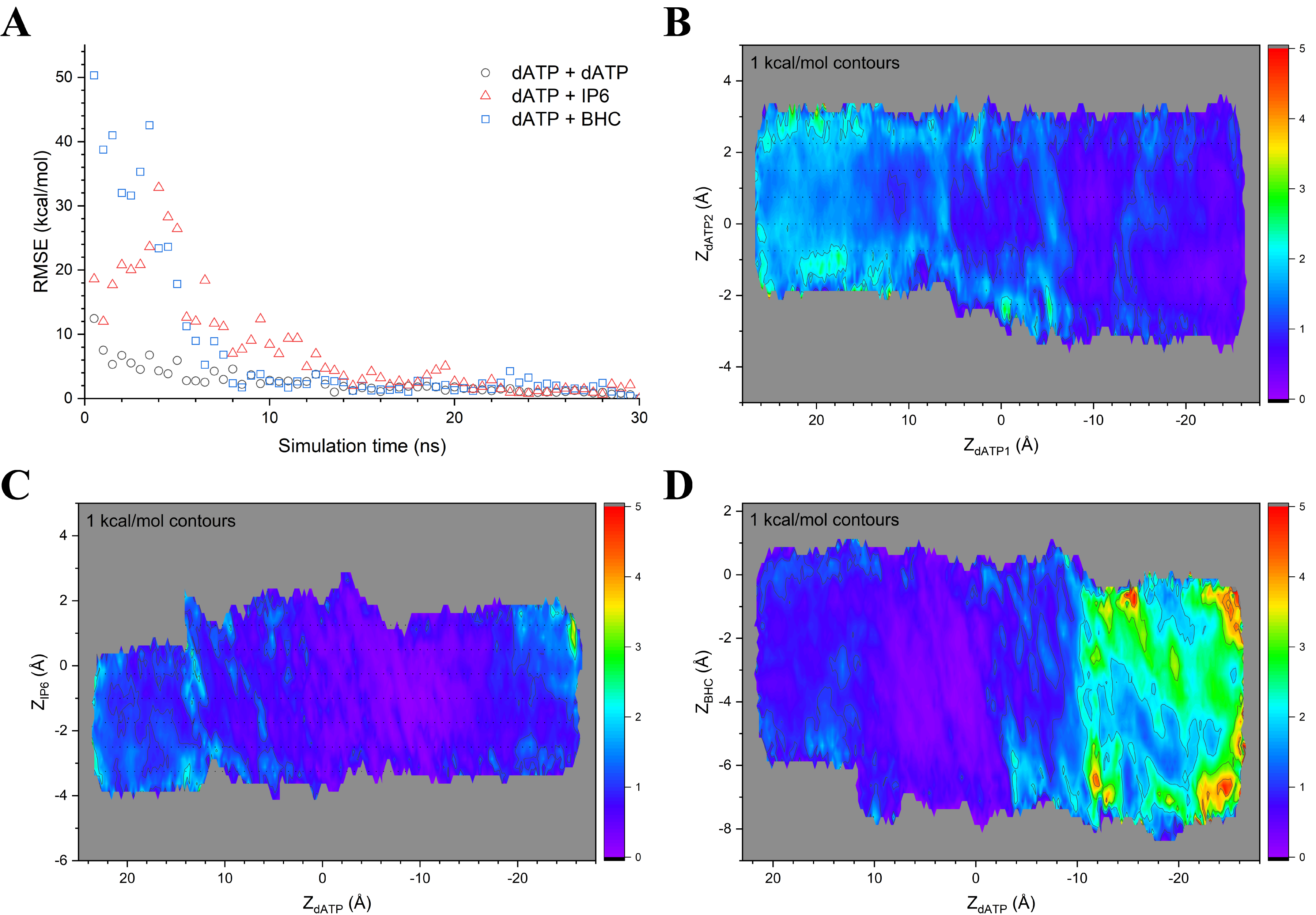

Supplement: S17 Fig — (A) Root mean squared errors of three 2D HREX/US simulations with 0.5 ns. Standard deviations of the 2D PMF surface of panel (B) the 2 dATP models, (C) dATP with IP6 and (D) dATP with BHC. Numerical data for panels A, B, C, and D can be found in the file S16 Data. BHC, benzenehexacarboxylic acid; dATP, deoxyadenosine triphosphate; HREX/US, Hamiltonian Replica-exchange/Umbrella Sampling; IP6, inositol hexakisphosphate; PMF, potential of mean force. (TIF) [file pbio.3001015.s017.tif]

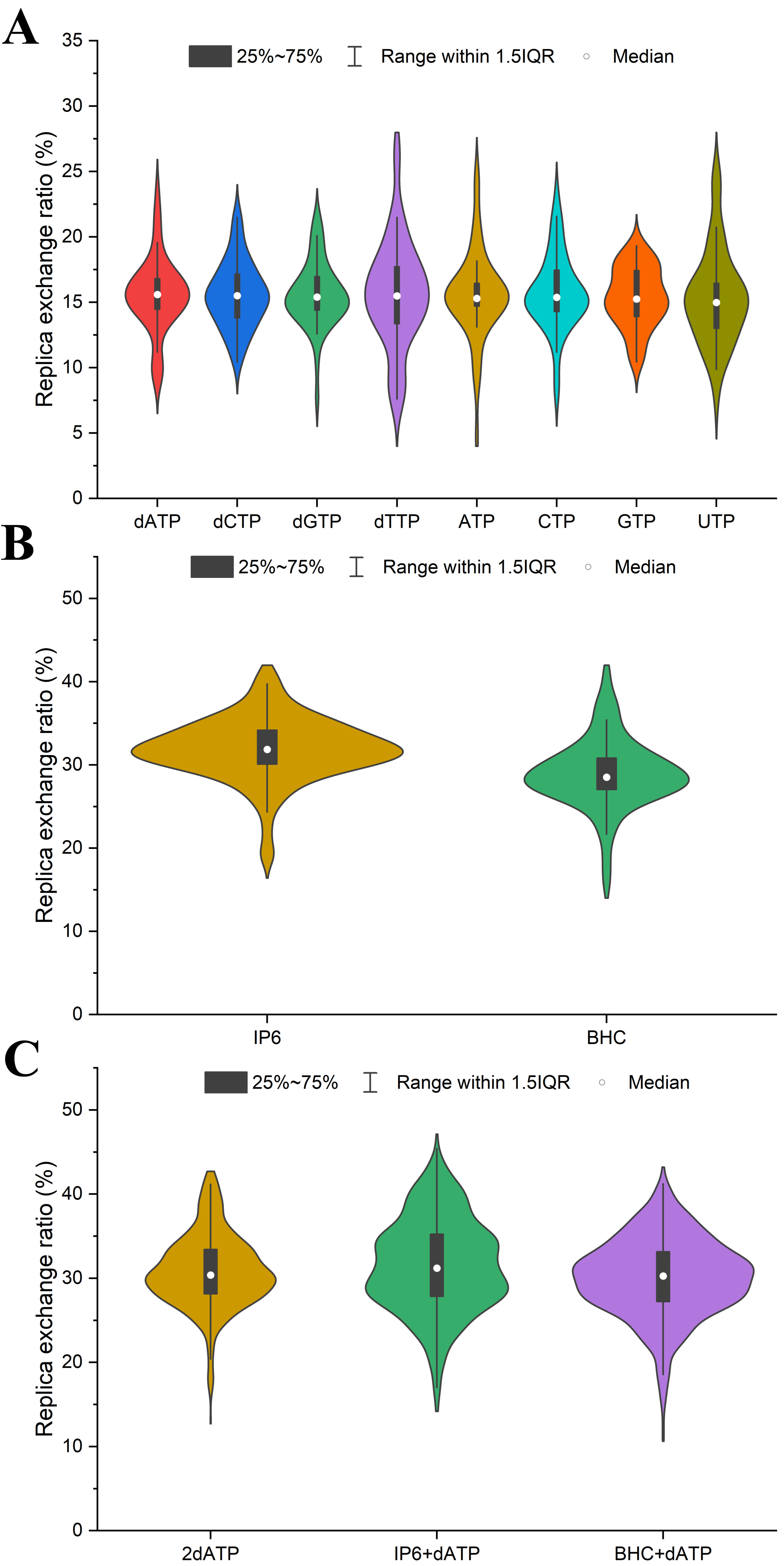

Supplement: S18 Fig — (A) 1D rNTP translocation simulation, (B) 1D IP6 and BHC translocation simulations, and (C) the three 2D dATP translocation simulations. Numerical data for panels A, B, and C can be found in the file S17 Data. BHC, benzenehexacarboxylic acid; dATP, deoxyadenosine triphosphate; HREX/US, Hamiltonian Replica-exchange/Umbrella Sampling; IP6, inositol hexakisphosphate; rNTP, ribonucleoside triphosphate. (TIF) [file pbio.3001015.s018.tif]

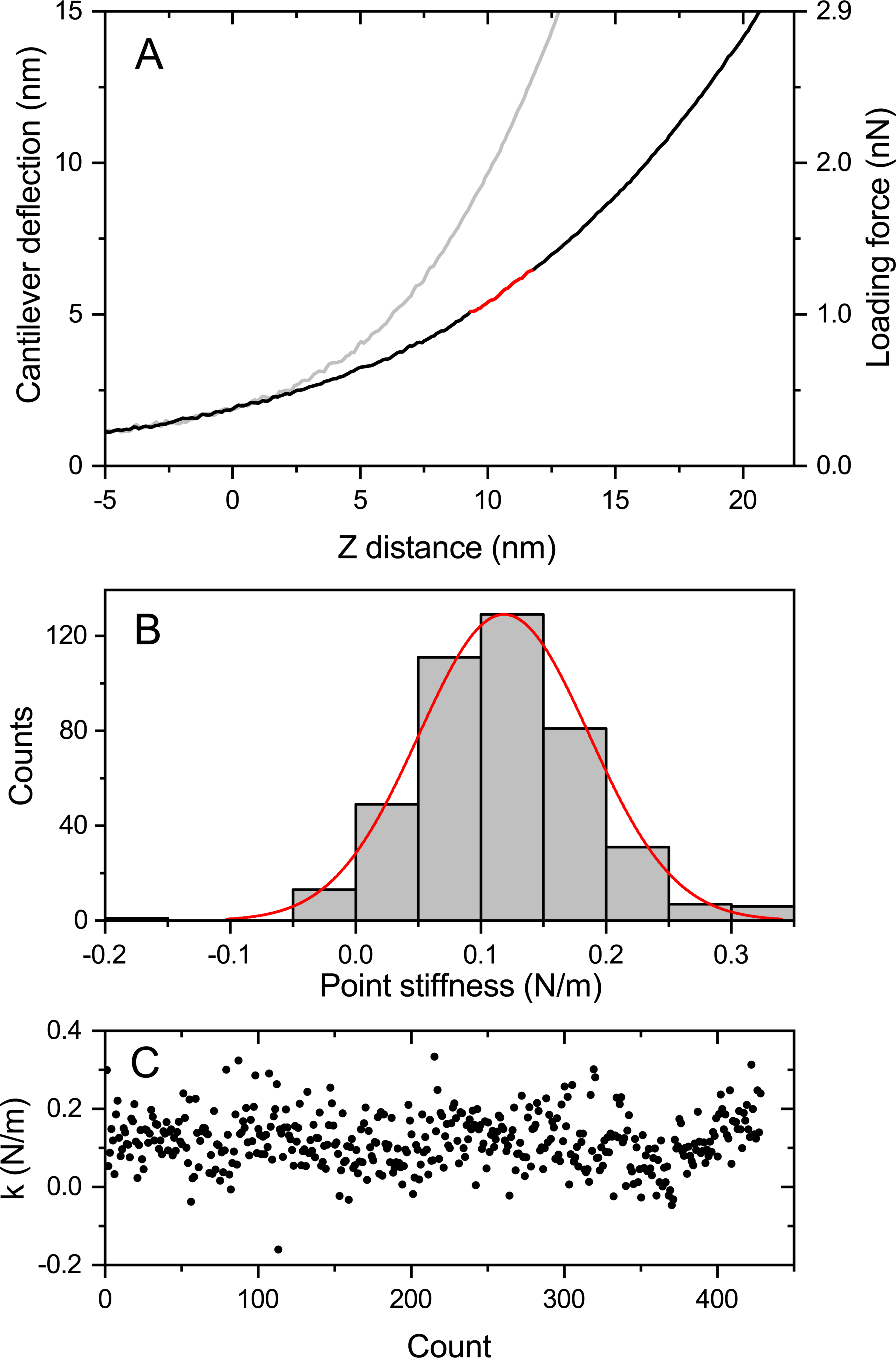

Supplement: S19 Fig — (A) Typical averaged force-distance curve of a core attached to an HMDS pretreated glass slide. For each experiment, approximately 400 such curves were acquired and averaged. Stiffness values were calculated by fitting a linear function to the force curve region bounded by 3- and 4-nm indentation depths. The corresponding line fit is plotted in red. (B) Histogram and Gaussian-fitted curves of the individual measured point stiffness values derived from the consecutive force-distance curves of a single core attached to a glass slide. (C) The individual measured point stiffness values obtained for the core shown in panel A, during a single experiment against the experiment number (count). The point stiffness measurements plot together with an analysis of the narrow distribution of the individual measured spring constants demonstrate that the core did not undergo a significant irreversible deformation during the indentation measurements. Numerical data for panels A, B, and C can be found in the file S18 Data. HMDS, hexamethyldisilazane. (TIF) [file pbio.3001015.s019.tif]

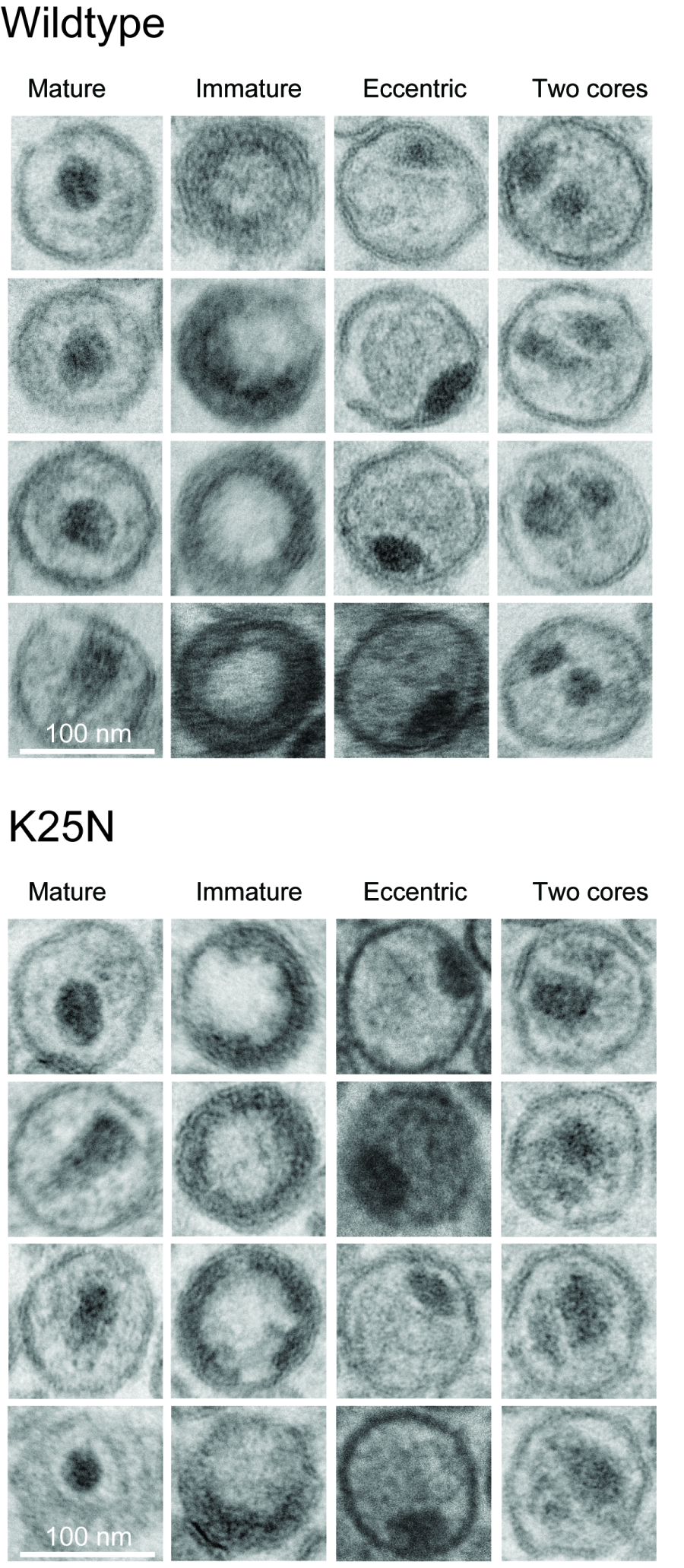

Supplement: S20 Fig — Magnification is ×30,000 (scale bar, 100 nm). The total number of WT, K25N, E45A, and K25N/E45A particles counted in each TEM experiment is shown in S7 Table. TEM, transmission electron microscopy; WT, wild-type. (TIF) [file pbio.3001015.s020.tif]

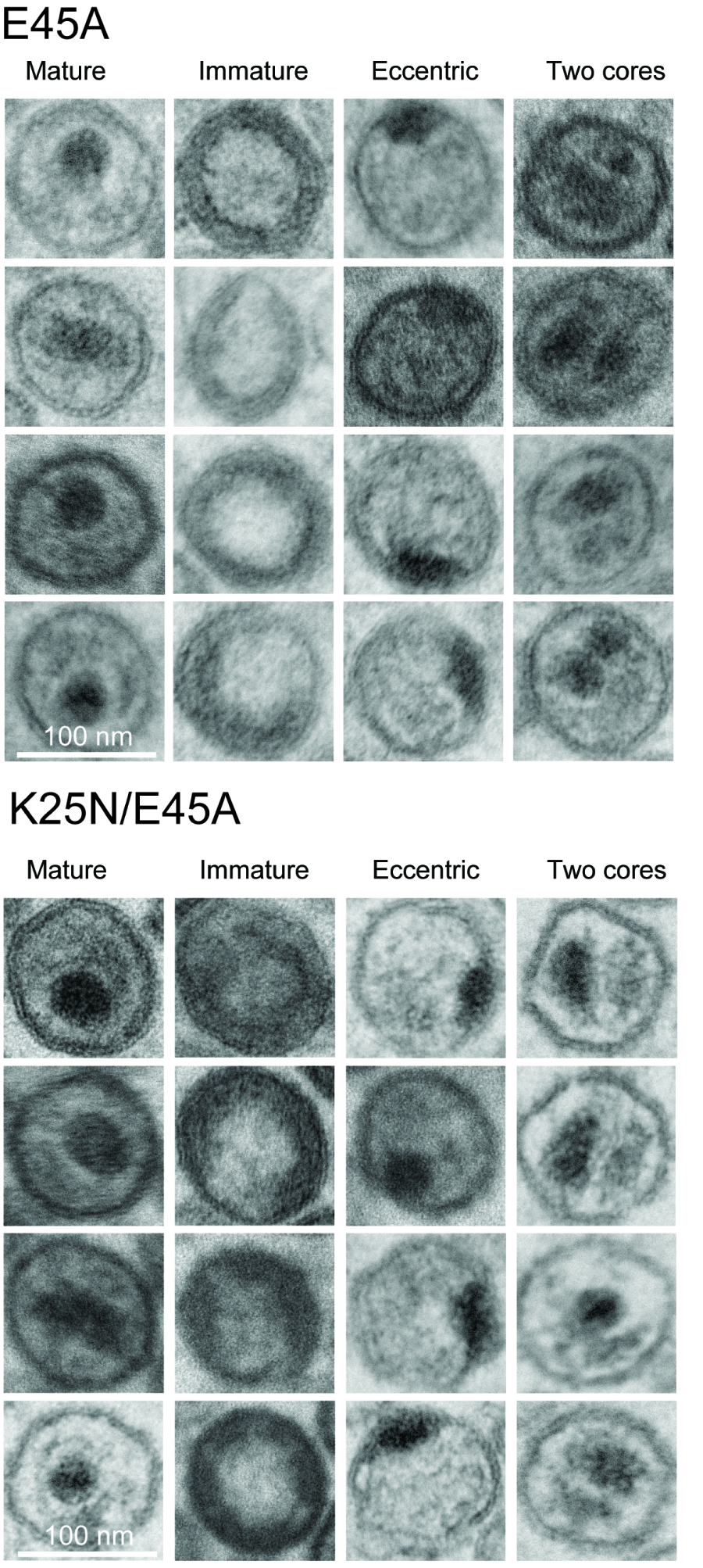

Supplement: S21 Fig — Magnification is ×30,000 (scale bar, 100 nm). The total number of WT, K25N, E45A, and K25N/E45A particles counted in each TEM experiment is shown in S7 Table. TEM, transmission electron microscopy; WT, wild-type. (TIF) [file pbio.3001015.s021.tif]
